# Marine Group II Euryarchaeota Contribute to the Archaeal Lipid Pool in Northwestern Pacific Ocean Surface Waters

**DOI:** 10.3389/fmicb.2020.01034

**Published:** 2020-06-05

**Authors:** Cenling Ma, Sarah Coffinet, Julius S. Lipp, Kai-Uwe Hinrichs, Chuanlun Zhang

**Affiliations:** ^1^State Key Laboratory of Marine Geology, Tongji University, Shanghai, China; ^2^Organic Geochemistry Group, MARUM Center for Marine Environmental Sciences and Department of Geosciences, University of Bremen, Bremen, Germany; ^3^Shenzhen Key Laboratory of Marine Archaea Geo-Omics, Southern University of Science and Technology, Shenzhen, China; ^4^Southern Marine Science and Engineering Guangdong Laboratory (Guangzhou), Guangzhou, China

**Keywords:** Marine Group II Euryarchaeota, Marine Group I Thaumarchaeota, Archaeal lipids, Ring Index, Northwestern Pacific Ocean, East China Sea

## Abstract

Planktonic archaea include predominantly Marine Group I Thaumarchaeota (MG I) and Marine Group II Euryarchaeota (MG II), which play important roles in the oceanic carbon cycle. MG I produce specific lipids called isoprenoid glycerol dibiphytanyl glycerol tetraethers (GDGTs), which are being used in the sea surface temperature proxy named TEX_86_. Although MG II may be the most abundant planktonic archaeal group in surface water, their lipid composition remains poorly characterized because of the lack of cultured representatives. Circumstantial evidence from previous studies of marine suspended particulate matter suggests that MG II may produce both GDGTs and archaeol-based lipids. In this study, integration of the 16S rRNA gene quantification and sequencing and lipid analysis demonstrated that MG II contributed significantly to the pool of archaeal tetraether lipids in samples collected from MG II-dominated surface waters of the Northwestern Pacific Ocean (NWPO). The archaeal lipid composition in MG II-dominated NWPO waters differed significantly from that of known MG I cultures, containing relatively more 2G-OH-, 2G- and 1G- GDGTs, especially in their acyclic form. Lipid composition in NWPO waters was also markedly different from MG I-dominated surface water samples collected in the East China Sea. GDGTs from MG II-dominated samples seemed to respond to temperature similarly to GDGTs from the MG I-dominated samples, which calls for further study using pure cultures to determine the exact impact of MG II on GDGT-based proxies.

## Introduction

Planktonic archaea are dominated by Marine Group I Thaumarchaeota (MG I; Brochier-Armanet et al., 2008; Spang et al., 2010) and Marine Group II Euryarchaeota (MG II; DeLong, 1992; Zhang et al., 2015). MG I are chemolithoautotrophs that use nitrification as a major energy acquiring mechanism (Könneke et al., 2005, 2014; Stahl and de la Torre, 2012) and occur predominantly in the deeper depths of open ocean, coastal seas, and estuaries (Karner et al., 2001; Teira et al., 2006; Caffrey et al., 2007; Schattenhofer et al., 2009; Xie et al., 2014; Nunoura et al., 2015; Sintes et al., 2015; Ingalls, 2016; Tian et al., 2018). Isolation, purification and laboratory cultures of MG I have enabled a better understanding of their physiology, biochemistry, and niche specification (Martens-Habbena et al., 2009; Qin et al., 2014; Bayer et al., 2015; Elling et al., 2015; Santoro et al., 2015).

Members of MG II have not been cultured; however, information on their lifestyle has been obtained through metagenomic studies. It is suggested that MG II live heterotrophically and occur mostly in the photic zone (Iverson et al., 2012; Zhang et al., 2015; Xie et al., 2018; Rinke et al., 2019; Santoro et al., 2019; Tully, 2019). However, MG II ecotypes have also been found in the deeper parts of the ocean (Li et al., 2015; Liu et al., 2017). Deep ocean MG II clades do not contain genes for proteorhodopsin, a light-driven protein present in MG II from the photic zone (Li et al., 2015). Other euryarchaeotal planktonic archaea include Marine Group III (MG III) that occur throughout the water column (Fuhrman and Davis, 1997; Haro-Moreno et al., 2017) and Marine Group IV (MG IV) that occur predominantly in the deep sea (López-García et al., 2001). MG III and MG IV are often present in low abundance. Little is known in terms of their ecological distribution and physiology (Santoro et al., 2019).

In parallel with molecular biology approaches, lipidomics is a powerful tool to study archaeal biogeochemical functions and adaptation to the environment. Notably, the development of high performance liquid chromatography – mass spectrometry (HPLC-MS) allows the identification of a wide range of archaeal membrane lipids (e.g., Schouten et al., 2000, 2008; Sturt et al., 2004; Becker et al., 2013; Wörmer et al., 2013; Zhu et al., 2013). Archaea possess unique membrane lipids: glycerol dibiphytanyl glycerol tetraethers (GDGTs) and archaeols (ARs; Koga and Morii, 2005; Koga and Nakano, 2008; Schouten et al., 2013). In living cells, thaumarchaeal lipids are dominated by intact polar lipids (IPLs) with monoglycosidic (1G-) or diglycosidic (2G-), phosphatidic (P-) or glycophosphatidic (HPH-) headgroups attached to the glycerol *sn*-1 hydroxyl position (Supplementary Figure S1). The core structure of GDGTs (C-GDGTs) may contain up to eight cyclopentane moieties (Supplementary Figure S1; de Rosa and Gambacorta, 1988; Kate, 1993; Schouten et al., 2013), one or two additional hydroxyl groups (OH-, 2OH-GDGTs; Supplementary Figure S1; Lipp and Hinrichs, 2009; Liu et al., 2012), or double bonds (unsaturated GDGTs with one to six double bonds; Zhu et al., 2014). AR containing a methoxy group at the *sn*-1 position of the glycerol moiety (MeO-AR) and macrocyclic archaeols (MARs) and unsaturated archaeols (uns-ARs; Zhu et al., 2013, 2016; Elling et al., 2014) are also observed in oceanic settings.

GDGTs contain information that can be used to evaluate paleo sea surface temperature (SST) (e.g., TEX_86_ index; Schouten et al., 2002), terrestrial organic matter input to the ocean (e.g., BIT index; Hopmans et al., 2004), or biogeochemical redox state in the ocean (e.g., Methane Index; Zhang et al., 2011). Although GDGTs have applications in paleoceanography and microbial ecology, their specific taxonomic sources remain ambiguous. Lipidomic studies on *Nitrosopumilus maritimus*, the first representative of MG I isolated from marine environments, reported lipids including GDGTs with zero to four cyclopentyl moieties and crenarchaeol, a GDGT containing one cyclohexyl and four cyclopentyl moieties (Könneke et al., 2005; Schouten et al., 2008). Crenarchaeol has so far only been observed in Thaumarchaeota (Sinninghe Damsté et al., 2002b; Elling et al., 2017) and has been postulated as a biomarker for archaeal nitrification (de la Torre et al., 2008; Pearson et al., 2008). Similarly, MeO-AR is reported to be present in all thaumarchaeal strains studied to date but does not appear to occur in crenarchaeal or euryarchaeal species. Thus, MeO-AR may also be used as a tentative biomarker for Thaumarchaeota (Elling et al., 2014, 2017).

Unsaturated acyclic archaeols (uns-ARs) with four double bonds were recently suggested as potential biomarkers for MG II based on analyses of uns-AR_0__:__4_ in suspended particulate matter (SPM) of epipelagic waters from the eastern tropical North Pacific, equatorial Pacific and off the coast of Cape Blanc (Zhu et al., 2016). Using a combination of GDGT analysis, metagenomics, and pyrosequencing of the SSU rRNA gene on samples from North Pacific Subtropical Gyre water column, it has been suggested that MG II also produce GDGTs, including crenarchaeol (Lincoln et al., 2014). This could potentially affect the use of TEX_86_, a SST proxy expressed as the ratio of GDGTs with different degree of cyclization (Schouten et al., 2002). TEX_86_ was proposed based on the premise that the large majority of GDGTs in the water column were solely produced by MG I (Schouten et al., 2002). Thus, the significant contribution of a second archaeal clade to the oceanic GDGT pool, as inferred by Lincoln et al. (2014), may complicate the relationship between TEX_86_ and SST. The findings of Lincoln et al. (2014) were debated by Schouten et al. (2014) who raised concern about the low abundance of extracted DNA and the use of C-GDGTs instead of IP-GDGTs, which are considered to better represent living biomass. Additional results were published by Besseling et al. (2020) who reported an absence of MG II-derived GDGTs from surface waters in the Atlantic Ocean and the North Sea. In addition, Zeng et al. (2019) identified two enzymes responsible for GDGT cyclization (i.e., GDGT ring synthases) and could only detect the related genes in metagenomes from MG I species and not in the MG II metagenomes. Together, these previous reports suggest that MG I Thaumarchaeota may be the dominant source of cyclized GDGTs in open ocean settings, although GDGT-producing MG II have been reported elsewhere (Wang et al., 2015). Therefore, the potential contribution of MG II to the GDGT pool in the ocean remains controversial.

In this study, we characterized and quantified archaeal membrane lipids in surface water samples from the Northwestern Pacific Ocean (NWPO) and East China Sea and supported these measurements with DNA sequencing and determination of cell density in order to determine the sources of the archaeal lipids. Both sample sets differed markedly in their archaeal community members with MG II being dominant in NWPO samples and MG I in East China Sea samples. Accordingly, the combined sample set was ideally suited to constrain the contribution of MG II to the marine archaeal lipid pool, to evaluate its effect on archaeal lipid based proxies, and to test previous hypotheses regarding candidate lipids of MG II (Lincoln et al., 2014; Zhu et al., 2016). In addition, this study presented the full range of intact and core archaeal lipids that were detected in surface waters, thus providing an important contribution towards a better understanding of the archaeal lipid distribution and related processes in the oceanic water column.

## Materials and Methods

### Shipboard Sampling and Environmental Parameters

Samples were collected on board the R/V *Dongfanghong II* during the East China Sea 2014 cruise (October; E-samples; The sampling plan and region were filed before the cruise started and approved by the Chinese Ministry of Foreign Affairs) and the NWPO 2015 spring cruise (April; N-samples). *In situ* temperature and salinity were measured by a conductivity-temperature-density (CTD) unit (Sea-Bird 911 CTD). Samples for inorganic nutrients (nitrate, nitrite, phosphate, and silicate) were filtered through 0.45 μm cellulose acetate filters and stored at −20°C until analysis with an AutoAnalyzer 3 HR (SEAL Analytical).

**FIGURE 1:**
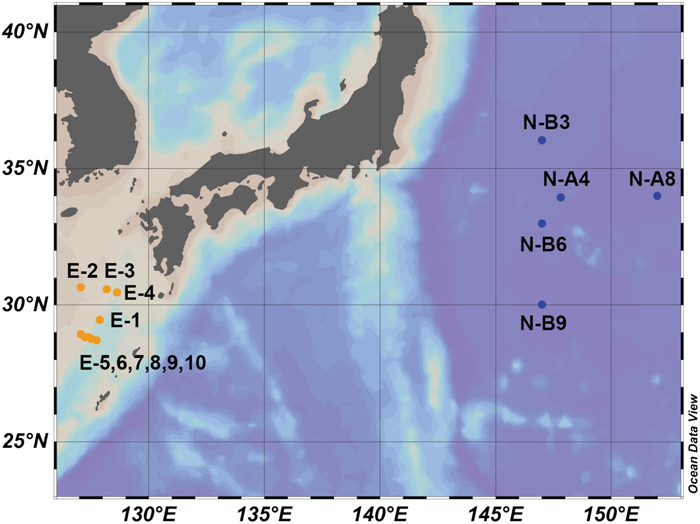
Map of sample collecting locations. Blue dots represent samples collected in Northwestern Pacific Ocean (NWPO) (N-samples). Orange dots represent samples collected in East China Sea (E-samples).

During each cruise, samples of SPM were collected from surface water (2 to 10 m; Figure 1 and Supplementary Table S1). For each sample, 80–200 L of seawater were filtered using a submersible pump through a GF/F filter (Whatman, 142 mm) of 0.7 μm pore diameter. Filters were then stored at −20°C until analysis. Previous studies (Ingalls et al., 2012) suggested that 0.7 μm GF/F filters may under-collect GDGTs in general and IP-GDGTs specifically because archaeal cells can be less than 1 μm (Könneke et al., 2005; Engelhardt, 2007). To ensure comparability, lipids and DNA were extracted from the same filters.

### Lipid Extraction

For each sample, lipids were extracted from 88% (7/8) of a freeze-dried GF/F filter. The filter was cut into slices and extracted using a modified Bligh and Dyer method (Zhang et al., 2013). In brief, the extraction was performed four times using methanol (MeOH), dichloromethane (DCM) and phosphate buffer at pH 7.4 (2:1:0.8 v/v). After sonication (10–15 min each time), additional DCM and buffer were added to achieve a final MeOH/DCM/buffer ratio of 1:1:0.9. The bottom organic phase was collected with a glass pipette (repeated at least three times). The total lipid extract was dried under N_2_, re-dissolved in MeOH and filtered using a 0.45 μm PTFE filter before analysis.

### Intact Polar and Core Lipid Analyses

Lipid separation was achieved on an ultra-high performance liquid chromatography (UHPLC) system (Dionex Ultimate 3000RS) in reversed phase conditions with an ACE3 C_18_ column (2.1 × 150 mm × 3 μm; Advanced Chromatography Technologies) maintained at 45°C (Zhu et al., 2013). Target compounds were detected by scheduled multiple reaction monitoring (sMRM) of diagnostic MS/MS transitions (Supplementary Table S2) on a triple quadrupole/ion trap mass spectrometer (ABSciEX QTRAP4500) equipped with a TurboIonSpray ion source operating in positive electrospray ionization (ESI) mode.

Quantification of lipids was achieved by adding an internal glycerol trialkyl glycerol tetraether standard (C_46_-GTGT; Huguet et al., 2006). Structures of the different lipids detected can be found in Supplementary Figure S1. Lipid abundance was corrected for response factors of commercially available as well as purified standards as described by Elling et al. (2014). In brief, abundances of IPLs were corrected by determining the response factors of purified 2G-GDGT-0 (for 2G-OH- and 2G-GDGTs), 1G-GDGT-0 (for HPH-, 1G-OH-, and 1G-GDGTs), 2G-AR (for 2G-AR) and 1G-AR (for 1G-AR) standards versus the C_46_-GTGT standard. Similarly, the abundance of C-GDGTs was corrected by the response factor of purified GDGT-0 standard versus the C_46_-GTGT standard. The abundances of C-AR, C-uns-ARs and MeO-AR were corrected by the response factor of a core archaeol standard (Avanti Polar Lipids, Inc. Alabaster, AL, United States) versus the C_46_-GTGT standard. In this study, C-uns-ARs are presented as C-uns-AR (u), where u = the number of double bond equivalents (DBE), representing either double bonds or rings and thus including both unsaturated and macrocyclic archaeols.

### Nucleic Acid Extraction, Quantitative Polymerase Chain Reaction, and Sequencing

DNA was extracted from 12% (1/8) of a GF/F filter (142 mm, ca. 4∼12 L filtered seawater) using the FastDNA SPIN Kit for Soil (MP Biomedical, Solon, OH, United States) with a final elution in 100 μL deionized water.

The archaeal 16S rRNA gene was quantified in all samples by quantitative polymerase chain reaction (qPCR; PIKO REAL 96, Thermo Fisher Scientific). Abundance (cells per liter) was normalized according to the dilution folds of DNA template and the volume of filtered seawater. The qPCR primers were Arch_787F (5′ ATTAGATACCCSBGTAGTCC 3′; Yu et al., 2005) and Arch_915R (5′ GTGCTCCCCCGCCAATTCCT 3′; Stahl, 1991).

Pyrosequencing was conducted on all samples, targeting the archaeal 16S rRNA gene. Primers were Arch_524F (5′ TGYCAGCCGCCGCGGTAA 3′) and Arch_958R (5′ YCCG GCGTTGAVTCCAATT 3′), which showed higher coverage for archaeal 16S rRNA gene as described recently (Cerqueira et al., 2017). Sequencing was performed using the Illumina Miseq platform at Majorbio Bio-Pharm Technology, Co., Ltd., Shanghai, China. Sequencing analysis was performed on the free online platform of Majorbio I-Sanger Cloud Platform^1^. Before analysis, sequences were demultiplexed and quality-filtered using QIIME (version 1.9.1). Sets of sequences with at least 97% identified were defined as an OTU (operational taxonomic unit), and chimeric sequences were identified and removed using UCHIME (Edgar et al., 2011). The taxonomy of each 16S rRNA gene sequence was analyzed using RDP Classifier^2^ against the SILVA ribosomal RNA gene database using a confidence threshold of 70% (Cole et al., 2009; Quast et al., 2013).

### Calculations and Statistical Analysis

Cell densities of MG I, MG II, and MG III were inferred based on total archaeal community composition derived from sequencing (Table 1) and total archaeal cell density obtained by qPCR (Table 2) according to equation 1 (MG I is taken as an example), where n = cell density (cells/L) and f = relative abundance (%):

(1) $$n_{M\,G\,I}=n_{t\,o\,t\,a\,l\,a\,r\,c\,h\,a\,e\,a}\times f_{M\,G\,I}$$

**TABLE 1:** Relative abundance of archaea based on archaeal 16S rRNA gene sequencing, of all detectable archaeal lipids and total archaeal lipid content based on UHPLC-MS analysis.

	**Relative abundance of archaea based on 16S rRNA gene sequencing (%)**			**Relative abundance of total archaeal lipids (%)**										**Total lipid content (ng/L)**
**Site**	**MG I**	**MG II**	**MG III**	**HPH-GDGTs**	**2G-OH-GDGTs**	**2G-GDGTs**	**1G-OH-GDGTs**	**1G-GDGTs**	**C-GDGTs**	**IP-ARs**	**C-AR**	**C-uns-ARs**	**MeO-AR**	
N-B3	5.2	94.3	0.5	0.5	6.6	9.4	0.4	21.0	60.6	0.2	0.1	0.7	0.4	12.0
N-A4	4.2	95.1	0.6	0.3	12.5	16.8	0.6	61.1	3.5	0.2	0.3	4.8	0.0	7.4
N-A8	2.6	94.9	2.4	1.4	13.3	26.0	0.7	44.6	8.6	0.3	0.5	4.6	0.1	4.1
N-B6	4.0	94.1	1.9	1.4	12.6	21.9	0.4	46.9	6.2	0.1	0.4	10.1	0.1	6.7
N-B9	0.2	99.7	0.1	0.1	11.6	19.4	0.7	45.6	19.4	0.1	0.1	2.7	0.1	2.9
Average of N-samples	3.3 ± 1.7	95.6 ± 2.1	1.1 ± 0.9	0.7 ± 0.6	11.3 ± 2.7	18.7 ± 6.2	0.5 ± 0.2	43.8 ± 14.4	19.7 ± 23.7	0.2 ± 0.1	0.3 ± 0.2	4.6 ± 3.5	0.1 ± 0.1	6.6 ± 3.5
Average of N-samples except N-B3	2.8 ± 1.6	96 ± 2.2	1.3 ± 0.9	0.8 ± 0.7	12.5 ± 0.7	21 ± 3.9	0.6 ± 0.2	49.5 ± 7.7	9.4 ± 7	0.2 ± 0.1	0.3 ± 0.2	5.6 ± 3.2	0.1 ± 0.03	5.3 ± 2.1
E-1	93.4	6.3	0.3	0.5	2.1	8.2	0	22.0	29.9	0.6	0.2	36.4	0.2	1.4
E-2	88.4	8.5	3.0	1.0	3.5	10.0	0	22.7	28.2	0.3	0.1	33.8	0.4	2.1
E-3	ND*	ND*	ND*	1.4	8.9	11.5	0.14	32.7	24.6	0.1	0.0	20.4	0.3	11.5
E-4	90.4	9.0	0.6	1.1	4.6	8.5	0.03	23.1	26.0	0.2	0.1	36.2	0.3	3.3
E-5	99.5	0.5	0.02	0.0	5.0	10.5	0.05	50.1	20.3	0.4	0.2	13.3	0.2	6.6
E-6	ND*	ND*	ND*	0.4	6.0	7.0	0.12	34.8	25.6	0.3	0.1	25.5	0.2	9.3
E-7	54.9	43.1	2.0	0.7	6.0	7.8	0.11	29.3	20.8	0.1	0.1	35.0	0.3	9.8
E-8	99.1	0.9	0.01	0.0	6.4	7.7	0.15	64.8	12.7	0.0	0.2	7.7	0.4	22.3
E-9	96.5	3.3	0.2	0.0	3.6	8.7	0.05	51.8	23.9	0.1	0.2	11.3	0.3	6.7
E-10	97.6	2.2	0.2	0.2	2.6	11.6	0.04	55.0	18.6	0.2	0.3	11.4	0.2	10.8
Average of E-samples	90 ± 13.8	9.2 ± 13.2	0.8 ± 1	0.5 ± 0.5	4.9 ± 2	9.1 ± 1.6	0.1 ± 0.06	38.6 ± 15.5	23 ± 5.1	0.2 ± 0.2	0.1 ± 0.1	23.1 ± 11.7	0.3 ± 0.1	8.4 ± 6.1

*^∗^ND, not detected.*

**TABLE 2:** Cell density of Archaea based on qPCR and cell densities of Marine Groups I, II, and III inferred by archaeal community composition from sequencing (the cell numbers are equivalent to gene copies assuming one cell contains one 16S rRNA gene of studied archaea); abundances of GDGTs, IP-GDGTs and intact polar crenarchaeol (IP-Cren, including HPH-, 2G- and 1G- crenarchaeol); cellular lipid contents in GDGTs and IP-GDGTs estimated for MG I and the whole archaeal cells.

	**Cell density (cells/L)**				**Lipid abundance (ng/L)**			**Cellular lipid content based on MG I cell density (fg/cell)**		**Cellular lipid content based on archaea cell density (fg/cell)**	
**Site**	**Archaea**	**MG I**	**MG II**	**MG III**	**GDGTs**	**IP-GDGTs**	**IP-Cren**	**GDGTs**	**IP-GDGTs**	**GDGTs**	**IP-GDGTs**
N-B3	4.4E + 07	2.3E + 06	4.2E + 07	2.3E + 05	11.8	4.6	1.0	5.16	1.99	0.27	0.10
N-A4	1.7E + 07	7.4E + 05	1.7E + 07	1.1E + 05	7.0	6.8	2.0	9.47	9.12	0.40	0.39
N-A8	5.1E + 06	1.3E + 05	4.8E + 06	1.2E + 05	3.9	3.5	0.8	28.8	26.2	0.76	0.69
N-B6	3.2E + 06	1.3E + 05	3.0E + 06	5.8E + 04	6.0	5.6	1.4	46.9	43.6	1.89	1.76
N-B9	7.4E + 06	1.7E + 04	7.3E + 06	8.5E + 03	2.8	2.2	0.7	165	132	0.38	0.30
E-1	1.9E + 07	1.7E + 07	1.2E + 06	6.3E + 04	0.9	0.5	0.2	0.05	0.03	0.05	0.02
E-2	2.3E + 07	2.0E + 07	2.0E + 06	7.0E + 05	1.4	0.8	0.3	0.07	0.04	0.06	0.03
E-3	2.0E + 07	ND*	ND*	ND*	9.1	6.3	2.4	ND*	ND*	0.45	0.31
E-4	3.6E + 07	3.3E + 07	3.2E + 06	2.1E + 05	2.1	1.2	0.5	0.06	0.04	0.06	0.03
E-5	4.0E + 07	3.9E + 07	2.0E + 05	6.7E + 03	5.7	4.3	2.3	0.14	0.11	0.14	0.11
E-6	2.5E + 07	ND*	ND*	ND*	6.9	4.5	2.1	ND*	ND*	0.27	0.18
E-7	1.3E + 07	7.1E + 06	5.6E + 06	2.6E + 05	6.3	4.3	1.6	0.89	0.61	0.49	0.33
E-8	3.9E + 07	3.9E + 07	3.5E + 05	5.7E + 03	20.5	17.7	8.8	0.53	0.45	0.52	0.45
E-9	5.5E + 07	5.3E + 07	1.8E + 06	1.3E + 05	5.9	4.3	2.5	0.11	0.08	0.11	0.08
E-10	5.7E + 07	5.6E + 07	1.3E + 06	9.6E + 04	9.5	7.5	4.3	0.17	0.13	0.17	0.13

*^∗^ND, not detected.*

The cellular lipid content (fg/cell) was calculated using the equations below for the MG I community only (Eq. 2) and for the whole archaeal community (Eq. 3), where n = cell density (cells/L) and a = lipid abundance (ng/L). Intact polar GDGTs (IP-GDGTs) were defined as the sum of HPH-, 2G-OH-, 2G-, 1G-OH-, and 1G-GDGTs. Total GDGTs are the sum of IP- and C- GDGTs.

(2) $$C\,e\,l\,l\,u\,l\,a\,r\,l\,i\,p\,i\,d\,c\,o\,n\,t\,e\,n\,t\,o\,f\,M\,G\,I=\frac{a_{t\,o\,t\,a\,l\,o\,r\,I\,P\,G\,D\,G\,T\,s}}{n_{M\,G\,I}}\times 10^{6}$$

(3) $$C\,e\,l\,l\,u\,l\,a\,r\,l\,i\,p\,i\,d\,c\,o\,n\,t\,e\,n\,t\,o\,f\,t\,o\,t\,a\,l\,a\,r\,c\,h\,a\,e\,a=\frac{a_{t\,o\,t\,a\,l\,o\,r\,I\,P\,G\,D\,G\,T\,s}}{n_{t\,o\,t\,a\,l\,a\,r\,c\,h\,a\,e\,a}}\times 10^{6}$$

Ring Index (RI) was calculated using Equation 4 according to Zhang et al. (2016):

(4) $$R\,I=\frac{1\times G\,D\,G\,T-1+2\times G\,D\,G\,T-2+3\times G\,D\,G\,T-3+4\times c\,r\,e\,n\,a\,r\,c\,h\,a\,e\,o\,l}{G\,D\,G\,T-0+G\,D\,G\,T-1+G\,D\,G\,T-2+G\,D\,G\,T-3+c\,r\,e\,n\,a\,r\,c\,h\,a\,e\,o\,l}$$

Cluster analysis in this study was performed by PAST software using the unweighted pair-group average algorithm. Correlation coefficients and *p*-values were obtained by analysis using R software. The neighbor-joining trees were constructed using MEGA software.

## Results

### Oceanographic Settings

The N-sample set was collected in the NWPO in April, 2015 and the E-sample set was collected in East China Sea in October, 2014. *In situ* temperature, salinity and nutrient content were determined for every sample. Salinity varied little between the two sample sets (Supplementary Table S1), but the temperature and nutrient contents of them changed substantially. The average *in situ* temperature of the N-samples was 18.1°C, with a range of 17.5–18.7°C. The E-samples had an average of 25.4°C, with a range of 24.3–26.4°C (Supplementary Table S1).

The fixed nitrogen contents in the N-samples were two to four times higher than those in the E-samples. The average nitrate content of the N-samples was 4.19 μmol/L with a range of 0.77 to 8.84 μmol/L (*n* = 5); the average nitrate content of the E-samples was 1.1 μmol/L with a range of 0.53 to 2.05 μmol/L (*n* = 10). The average nitrite content of the N-samples was 0.16 μmol/L with a range of 0.02 to 0.27 μmol/L, while that of the E-samples was 0.04 μmol/L with a range of 0.01 to 0.14 μmol/L.

Phosphate content averaged 0.11 μmol/L (ranging from 0.03 to 0.2 μmol/L, *n* = 5) in the N-samples and 0.05 μmol/L (ranging from 0.03 to 0.08 μmol/L, *n* = 10) in the E-samples. Silicate content averaged 2.45 μmol/L (ranging from 0.11 to 4.68 μmol/L) in the N-samples and 0.95 μmol/L (ranging from 0.24 to 1.57 μmol/L) in the E-samples (Supplementary Table S1).

### Archaeal Community Structure

**FIGURE 2:**
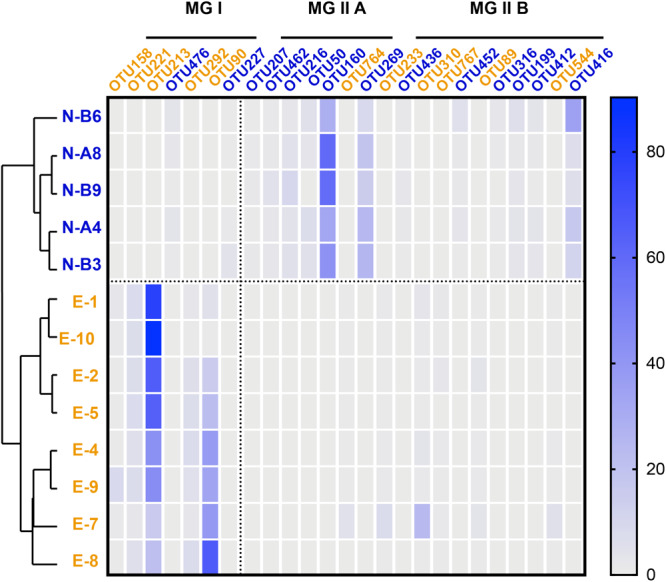
Heatmap based on the distribution of representative archaeal OTUs (labels marked in blue represent major OTUs from N-samples; labels in orange represent OTUs from E-samples). Color scale on the right correspond to the percentage of OTU 16S rRNA gene amplicon reads over the total reads. Samples are listed in the order of OTU-distribution cluster analysis result (blue labels are N-samples and orange labels are E-samples).

Results of the 16S rRNA gene sequencing showed that archaeal communities substantially differed between the N- and E- sample sets. In the N-samples collected in April 2015 (Figure 1), the relative abundance of MG I ranged from 0.2 to 5.2%. MG II represented the vast majority of 16S rRNA gene reads with a range from 94.1 to 99.7% (Table 1 and Figure 2). In contrast, in most E-samples collected in October 2014 (Figure 1), MG I accounted for more than 88.4% while MG II accounted for less than 9.0% (except E-7 with MG I accounting for 54.9% and MG II accounting for 43.1%; Table 1 and Figure 2). For both sets of samples, MG III only accounted for a small proportion of archaea, from 0.01 to 3.0% and other unclassified archaea accounted for less than 3.0% of the total archaeal sequences at all stations. Hence, we normalized the sequencing results by setting the sum of MG I, II and III to 100% (Table 1).

Seven most prevalent archaeal OTUs were assigned to MG I and 18 to MG II (7 to MG II A and 11 to MG II B; Figure 2 and Supplementary Figure S3). Among MG I, OTU 476 was predominant in N-samples (occupied over 82.7% in MG I and 1.8 ± 1.3% in total reads). An exception was sample N-B3 that showed a predominance of OTU 227 occupying 88.0% in MG I and 1.0 ± 1.5% in total reads. OTU 213 and OTU 90 were predominant in E-samples (averaging 56.0 ± 24.0 and 31.3 ± 23.7% in MG I, 52.6 ± 24.9 and 27.1 ± 19.9% in total reads, respectively; Figure 2).

Among MG II, OTU 160 and OTU 269 were predominant in N-samples (affiliated to MG II A; averaging 45.0 ± 12.9 and 18.6 ± 6.4% in MG II, 44.0 ± 13.0 and 18.0 ± 6.1% in total reads, respectively). OTU 310, OTU 89 and OTU 233 had higher relative abundance in E-samples (affiliated to MG II B; averaging 40.9 ± 13.2, 19.6 ± 12.9, and 14.2 ± 6.4% in MG II; 4.1 ± 7.2, 1.0 ± 1.2, and 1.2 ± 2.3% in total reads; Figure 2 and Supplementary Figure S3).

### Archaeal Cell Density and Total Lipid Content

Archaeal cell density in each sample was quantified by qPCR targeting the archaeal 16S rRNA gene. On average, 1.5 × 10^7^ archaeal cells per liter of seawater (cells/L; Table 2) were observed in N-samples (ranging from 3.2 × 10^6^ to 4.4 × 10^7^ cells/L). E-samples showed a slightly higher average archaeal cell density of 3.3 × 10^7^ cells/L (Table 2).

MG I and MG II specific cell densities were estimated by multiplying their relative abundance within total archaeal sequences with the qPCR-derived archaeal cell density (Eq. 1). As a result, MG I ranged between 1.7 × 10^4^ and 2.3 × 10^6^ cells/L in N-samples and between 7.1 × 10^6^ and 5.6 × 10^7^ cells/L in E-samples. MG II in N-samples were 1–2 orders of magnitude higher (3 × 10^6^ to 4.2 × 10^7^ cells/L) than in E-samples (2 × 10^5^ to 5.6 × 10^6^ cells/L; Table 2 and Supplementary Figure S2).

The total archaeal lipid content varied greatly within each sample set. Total archaeal lipids ranged from 2.9 to 12 ng/L (average was 6.6 ± 3.5 ng/L) in N-samples and from 1.4 to 22.3 ng/L (average was 8.4 ± 6.1 ng/L) in E-samples (Table 1).

### Archaeal Lipid Distribution

**FIGURE 3:**
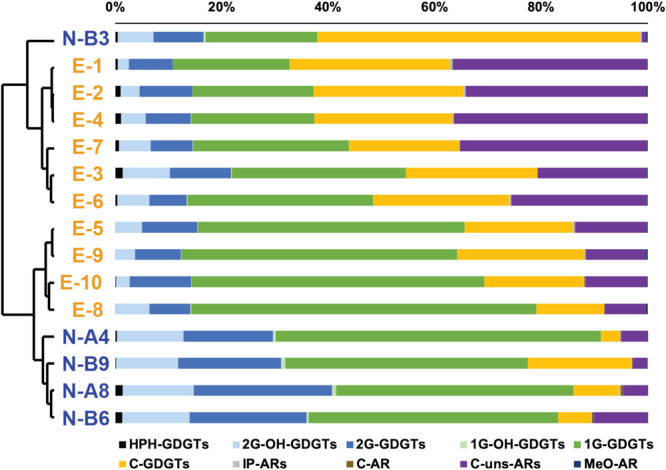
Cluster analysis based on the distribution of all detectable archaeal membrane lipids. Chemical structures are shown in Supplementary Figure S1. Blue labels are N-samples and orange labels are E-samples.

Except for sample N-B3 (characterized by a predominance of C-GDGTs), all N-samples were dominated by 1G-GDGTs followed by 2G-GDGTs and C-GDGTs (Figure 3 and Table 1). These three components were also the major lipids in E-samples. In both N- and E- samples, 2G-OH-GDGTs with 0–4 cyclopentyl rings were detected, in which 2G-OH-GDGTs -3 and -4 have not been reported in previous studies. These two compounds were identified based on exact mass and retention pattern with the sMRM method and also with a parallel quadrupole time-of-flight tandem mass spectrometer (qTOF-MS) analysis (Supplementary Table S2; cf. Zhu et al., 2013). 2G-OH-GDGTs relative abundance ranged between 6.6 and 13.3% in the N-samples and between 2.1 and 8.9% in the E-samples. HPH-GDGTs accounted for less than 2% of total archaeal lipids in every sample and 1G-OH-GDGTs (including 1G-OH-GDGTs -0, -1, and -2) for less than 1% (Table 1). GDGTs with 0–3 cyclopentyl rings and crenarchaeol were detected (see chemical structures in Supplementary Figure S1 and relative abundances in Supplementary Table S3) but the crenarchaeol isomer, unsaturated GDGTs and OH-GDGTs were not detected. The ring distribution of 2G-GDGTs was different from those of HPH-, 1G-, and C- GDGTs. The former group was dominated by GDGTs -1, -2, and -3 while GDGT-0 and crenarchaeol predominated in the latter (Supplementary Table S3).

ARs accounted for 1.4 to 37.3% of total archaeal lipids (Table 1) with core unsaturated ARs (C-uns-ARs, with 1 to 7 DBE) being the most abundant AR types (Table 1 and Supplementary Figure S1). IP-ARs (including 1G- and 2G- AR), C-AR, and methoxy archaeol (MeO-AR) all represented less than 1% of total archaeal lipids in every sample (Table 1 and Figure 3).

A cluster analysis performed on the comprehensive lipid distribution showed distinct lipid distributions between the N- and E- samples. The N-B3 sample had a significantly different lipid distribution from other samples and was thus subsequently considered as an outlier. Lipid distributions of N- and E- samples mainly differed upon the relative abundance of C-GDGTs, 2G-GDGTs, 1G-GDGTs, 2G-OH-GDGTs, and C-uns-ARs (Figure 3). In MG I-dominated E-samples, 1G-GDGTs ranged from 22 to 64.8% (38.6 ± 15.5% in average), C-GDGTs from 12.7 to 29.9% (23 ± 5.1% in average), and 2G-GDGTs from 7 to 11.6% (9.1 ± 1.6% in average; Table 1). By contrast, in MG II-dominated N-samples, lipid distribution showed higher relative amounts of 1G-GDGTs (49.5 ± 7.7% in average), 2G-GDGTs (21 ± 3.9% in average) and lower amounts of C-GDGTs (9.4 ± 7% in average; Table 1). Among the minor lipids, the E-samples showed higher amounts of C-uns-ARs (23.1 ± 11.7% in average) while the N-samples had a higher relative abundance of 2G-OH-GDGTs (12.5 ± 0.7% in average; Figure 3 and Table 1).

## Discussion

### Potential Contribution of MG II Euryarchaeota to the Archaeal Tetraether Lipid Pool

Previous studies showed that MG II usually inhabit the surface photic zone while MG I are found in deeper layers of the water column (e.g., Zhang et al., 2015; Ingalls, 2016), which is consistent with the observed microbial community in the N-samples but not in the E-samples. Seasonality may explain the observed contrasted community structure between the two sample sets as it was previously observed to impact MG I (Massana et al., 1997; Murray et al., 1998; Galand et al., 2010; Hollibaugh et al., 2011; Pitcher et al., 2011b) and MG II (Pernthaler et al., 2002; Galand et al., 2010) communities. Previous studies reported MG I blooms during low phytoplanktonic productivity season (Pitcher et al., 2011a), which is consistent with the predominance of MG I in the E-samples collected in October (He et al., 2013). In contrast, the MG II-dominated samples (N-samples) were sampled in spring. This is in agreement with the detection of a spring MG II bloom in surface waters at German Bight in the North Sea (Pernthaler et al., 2002). Besides, we observe that the predominant MG II OTUs (OTU 160 and OTU 269; Figure 2 and Supplementary Figure S3) in the N-samples collected in April are phylogenetically affiliated to MG II A, which were previously observed to be predominant in summer when nutrients become depleted. On the contrary, MG II B seem to be more abundant during winter when nutrients are replenished (Galand et al., 2010).

The unambiguous difference in archaeal community structure between the two sample sets offers the opportunity to analyze in detail the lipid contribution by the uncultivated MG II archaea in the samples they dominate. For this purpose, IPLs were analyzed from the same filters used for archaeal community analysis. IPLs, particularly those with phosphate head groups, are assumed to be rapidly degraded upon cell death (White et al., 1979; Harvey et al., 1986) and hence are representative of the active living archaeal community at the moment of sampling. We thus consider the overprint from extinct cell biomass to be minimal.

**FIGURE 4:**
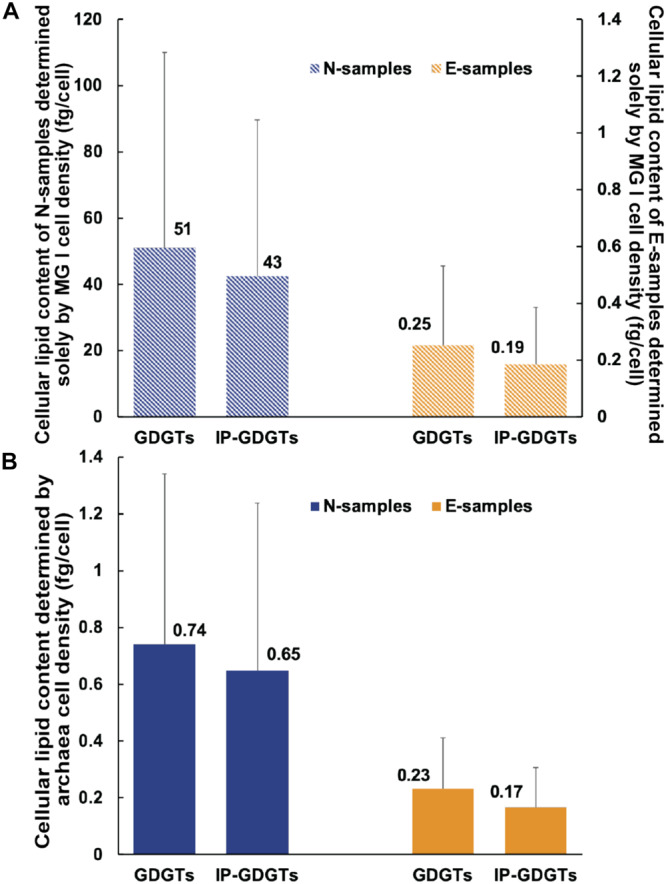
Variation of inferred cellular lipid contents: **(A)** Assuming MG I being the sole source of GDGTs and **(B)** assuming archaea including MG I and MG II being the source of GDGTs. The cellular lipid content was calculated by dividing the lipid concentrations measured from UHPLC-MS by the cell numbers (MG I or total archaea) estimated from qPCR and sequencing. Blue columns represent the average value of the N-samples. Orange columns represent the average value of the E-samples. Bars represent standard deviation in each sample set. In Figure **(A)** the primary y axis on the left represents values of the N-samples and secondary y axis on the right represents values of the E-samples. The scale of the primary y axis is different from the secondary y axis. In Figure **(A)**, the bars are hatched to highlight that these values are hypothetical.

MG I are considered as the dominant source of IPL-GDGTs in the ocean (Schouten et al., 2013). In this study, we determined how much lipid a MG I cell would contain if all detected GDGTs were exclusively produced by MG I cells (Eq. 2). In the E-samples, cellular GDGT content varies from 0.05 to 0.89 fg/cell (0.25 fg/cell in average; Table 2 and Figure 4A) and cellular IP-GDGT from 0.03 to 0.61 fg/cell (0.19 fg/cell in average; Table 2 and Figure 4A) for MG I. These values are close to previous estimates of MG I cells based on both environmental and pure culture samples (1 fg/cell, Sinninghe Damsté et al., 2002b; 0.25 fg/cell, Schouten et al., 2012; 0.9 to 1.9 fg/cell, Elling et al., 2014).

In the MG II-enriched N-samples, assuming all GDGTs derive from MG I cells, the calculated cellular archaeal lipid quota would be 5.16 to 165 fg/cell (51 fg/cell in average) for total GDGTs and 1.99 to 132 fg/cell for IP-GDGTs (43 fg/cell on average; Table 2 and Figure 4A). These values are two orders of magnitude higher than the results found in the E-samples as well as in former studies. We hypothesize that the overestimation of cellular lipid content in the N-samples may be due to neglecting GDGT production from the MG II communities as previously suggested by Lincoln et al. (2014) in the North Pacific Subtropical Gyre shallow and intermediate depths. Consequently, cellular lipid contents in total GDGTs and IP-GDGTs were calculated based on the total archaeal cell density (Eq. 3; Table 2). Total cellular GDGT content in the N-samples then ranges from 0.27 to 1.89 fg/cell (0.74 fg/cell on average) and cellular IP-GDGT content from 0.1 to 1.76 fg/cell (0.65 fg/cell on average; Table 2 and Figure 4B). These values are in the same order of magnitude as the estimates from the E-samples as well as from previous studies. Therefore, the observed GDGT distributions can be most plausibly explained by the production of GDGTs by MG II community members.

Our observations are inconsistent with those made in a recent study (Besseling et al., 2020), which estimated the potential contribution of MG II to the IPL pool by analysis of (sub)surface waters of the North Atlantic Ocean and the coastal North Sea. These authors did not detect IP-GDGTs and other archaeal IPLs in samples dominated by MG II and concluded that MG II contributed neither to GDGTs nor to any other known archaeal IPLs (Besseling et al., 2020). Nonetheless, potential alternative lipids belonging to the abundant MG II were not identified. Currently, we can only speculate that the inconsistency between our study and theirs may be due to geographical difference or different quantification approaches. Ultimately the analysis of an MG II isolate, when available, will shed more light on the lipid composition for archaeal lipids of this ubiquitous planktonic archaeal group.

### Differences of Lipid Distribution Between MG I and MG II Enriched Sample Sets

Cluster analysis of the lipidomic distributions suggests significant differences between the N- and E- samples, which further supports a potential contribution from MG II to the lipid pool (Figure 3). MG I-enriched E-samples show high abundances of 1G-GDGTs, 2G-GDGTs and C-GDGTs (Figure 3), which is consistent with former studies in MG I-enriched marine environments (Schouten et al., 2008; Pitcher et al., 2011b; Sinninghe Damsté et al., 2012). Besides, Elling et al. (2017) comprehensively described the lipid inventory of 10 Thaumarchaeal cultures in which members of Group 1.1a, inhabiting marine environments, were characterized by high abundances of 1G-GDGTs, 2G-GDGTs and 2G-OH-GDGTs (in decreasing order of abundance). MG II-dominated N-samples exhibit lower amounts of C-GDGTs and increased amounts of 1G-GDGTs, 2G-GDGTs and 2G-OH-GDGTs. Higher relative abundance of 2G-GDGTs in MG II-dominated N-samples is consistent with the observation in the Mediterranean Sea water column, where MG II were positively correlated with 2G-GDGTs (Besseling et al., 2019).

In addition, we observed high abundances of C-uns-ARs in MG I-enriched E-samples (between 7.7 and 36.4%; 23.1 ± 11.7% in average) and low values in the N-samples (between 2.7 and 10.1%; 5.6 ± 3.2% in average; Table 1). Hence, C-uns-ARs may potentially be biomarkers for MG I, as also demonstrated by significant correlations between MG I cell density and abundances of C-uns-ARs (except outlying sample E-7; Supplementary Figure S4a). However, C-uns-ARs have never been observed in pure cultures of MG I as well as in other Thaumarchaeota (Elling et al., 2017). Instead, other studies attributed C-uns-ARs to MG II or methanogen sources (Zhu et al., 2016; Baumann et al., 2018). Specifically, Zhu et al. (2016) observed that C-uns-AR_0__:__4_ was particularly abundant in the euphotic zone of the Equatorial Pacific. Baumann et al. (2018) reported C-MARs in two strains of (hyper)thermophilic methanogens. Thus, these compounds may be produced by a large range of Archaea, including both MG I and MG II. Furthermore, both in the N- and E- sample sets, C-uns-AR (4) dominated, followed by C-uns-AR (6), C-uns-AR (5), and C-uns-AR (3) (Supplementary Table S5). Accordingly, the unsaturation degree of C-uns-ARs showed little variation between the N- and E- sample sets (Supplementary Table S5). This suggests that the contrasting temperature and archaeal community structure between the two sample sets have little effect on the unsaturation degree of C-uns-ARs in this study.

Previous studies of SPM from surface water identified HPH-GDGTs, especially HPH-crenarchaeol as produced by MG I (Besseling et al., 2019; Sollai et al., 2019). In this study, HPH-GDGTs are detected in every sample as a minor constituent (less than 1.5%); however, HPH-crenarchaeol systematically shows higher relative abundance in MG I-dominated E-samples than in MG II-dominated N-samples (Supplementary Table S3), further supporting potential chemotaxonomic specificity of HPH-crenarchaeol for MG I. Among core lipids, crenarchaeol (Sinninghe Damsté et al., 2002a, b; Schouten et al., 2013) and MeO-AR (Elling et al., 2014, 2017) were both postulated as biomarkers for MG I. We do not observe any correlation between MG I and crenarchaeol in the present data set, suggesting that core crenarchaeol does not appear to be a universal biomarker for MG I (Supplementary Table S4). Instead, core crenarchaeol correlates with MG II cell density (Supplementary Table S4). This is consistent with the positive correlations between the abundance of specific MG II subgroups with HPH-crenarchaeol and 2G-crenarchaeol observed in the Mediterranean Sea water column (Besseling et al., 2019). But this is inconsistent with the genome-mining results suggesting that MG II do not contain the recently discovered genes encoding the enzymes responsible for ring insertions in GDGTs (Zeng et al., 2019). However, Zeng et al. (2019) also noted that MG II from natural environments may use other pathways for GDGT synthesis (including ring structures), which have yet to be characterized. MeO-AR in both the N- and E- samples shows low absolute abundance (relative abundances less than 0.5%; Figure 3 and Table 1). The relative abundance of MeO-AR slightly increases in MG I-enriched E-samples (Figure 3 and Table 1) but there is no significant correlation between MG I cell density and MeO-AR abundance (Supplementary Figure S4b). Accordingly, its reliability as diagnostic MG I biomarker is also questionable.

Based on the discussion above, no specific biomarkers for MG II could be identified. Previous findings based on genome analysis suggested that MG II may have the potential to synthesize mixed membranes consisting of archaeal type ether lipids with bacterial/eukaryotic G3P glycerol-phosphate backbones (Villanueva et al., 2016; Rinke et al., 2019). However, our results demonstrate a circumstantial link between the existence of the commonly found GDGTs and the dominance of MG II in the NWPO where MG I were present at less than 5.2% of the total archaeal community.

### Temperature Is the Main Driver for Archaeal Lipid Distribution in Samples With Different Archaeal Communities

**FIGURE 5:**
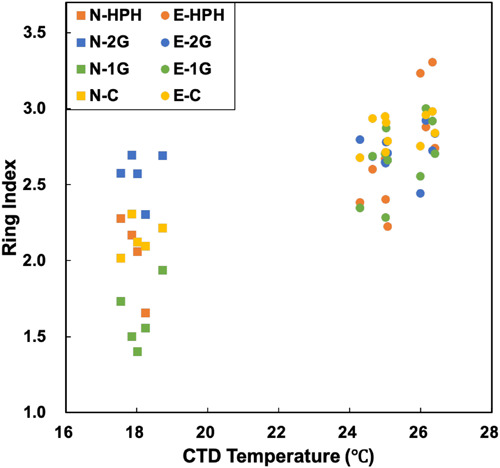
Plot of *in situ* temperature against the ring index. Significant correlations exist in 1G- and C- GDGTs, with *R*^2^ value of 0.89 and 0.92 (*p-*value < 0.001, regression lines are not shown), respectively.

The TEX_86_ proxy was developed to reconstruct past SSTs based on GDGT distribution and is now being regularly used in paleoceanography studies (Schouten et al., 2002, 2013; Tierney and Tingley, 2015). The prerequisite for this proxy is that temperature should be the main driver of GDGT distribution in the environment. It was indeed demonstrated in culture experiments on archaeal strains that higher temperatures led to higher cyclization degree (Uda et al., 2001, 2004; Lai et al., 2008; Boyd et al., 2011). However, several studies pointed to additional environmental factors which could also influence GDGT cyclization degree. For instance, TEX_86_ values increase in late growth phases (Elling et al., 2014), at lower O_2_ concentrations (Qin et al., 2015) and with lower ammonia oxidation rate (Hurley et al., 2016; Evans et al., 2018). Besides, archaeal community structure is known to have an effect on GDGT distribution (Wuchter, 2006; Herfort et al., 2007; Turich et al., 2007; Elling et al., 2015). In this study, no crenarchaeol isomer was detected precluding the calculation of TEX_86_. The ring index (RI), which behaves similarly to TEX_86_ (Schouten et al., 2002), was thus computed in order to estimate the potential impact of MG II-produced GDGTs on the TEX_86_ proxy (Figure 5; Eq. 4; Zhang et al., 2016).

The Ring Index of 1G- and C- GDGTs shows strong correlations with *in situ* temperature, with lower RI corresponding to lower *in situ* temperatures in MG II-dominated N-samples and higher RI to higher *in situ* temperatures in MG I-dominated E-samples (Figure 5 and Supplementary Table S6). This implies that *in situ* temperatures apparently influenced the RI of these lipid pools in both sample sets with different archaeal communities.

The low sensitivity of 2G-RI to temperature indicates that the cyclization degree of 2G-GDGTs may be less impacted by temperature. 2G-GDGTs are dominated by GDGTs -2 and -3 and crenarchaeol, while the other GDGT pools show higher abundances of crenarchaeol and GDGT-0 (Supplementary Table S3). In addition, the MG II-dominated N-samples show higher abundances of 2G-GDGTs (Table 1). The lack of correlation between 2G-RI and temperature may suggest a potentially high impact of the planktonic archaeal community structure on archaeal temperature reconstruction proxies. Indeed, our results suggest that (i) MG II may be significant contributors of 2G-GDGTs (Figure 3 and Table 1) and (ii) 2G-GDGT cyclization degree is only weakly impacted by temperature. These data call for further investigation aiming at determining (i) the global contribution of MG II to the archaeal lipid pool produced in the water column and (ii) the export mechanisms of IPLs and particularly 2G-GDGTs to the seafloor. Understanding these two key points are of prime importance for evaluating the potential impact of MG II communities on the TEX_86_-based paleotemperature proxies.

## Conclusion

16S rRNA gene sequencing results of two sample sets collected from surface waters of NWPO and East China Sea highlighted substantial differences in archaeal community structures between the two sample sets, with MG II dominating the former and MG I the latter. By examining the absolute lipid abundance and archaeal cell densities estimated from qPCR and sequencing analysis, we revealed a potentially high contribution of MG II to archaeal tetraether lipids in MG II-dominated samples collected from surface waters of the NWPO. This is consistent with an early observation of MG II contribution to the GDGT pool in the North Pacific Subtropical Gyre. Archaeal lipid distribution in these samples differed from the MG I-dominated samples collected from East China Sea surface waters. Notably, higher abundances of unsaturated archaeols were observed in the MG I-dominated samples than in the MG II-dominated ones. The widespread occurrence of these unsaturated compounds implies that they may be synthesized by a large range of Archaea. However, the lipid distribution differences seemed to only marginally impact the cyclization degree of the whole GDGT pool produced in the surface waters. Overall, this study provides new clues on the distribution of MG II archaeal lipids and their biological sources in oceanic surface water, while cautioning the use of archaeal lipid-based proxies for paleoclimate reconstruction.

## Data Availability Statement

The 16S rRNA gene amplicon reads (raw data) in this study have been deposited in the NCBI Sequence Read Archive (SRA) under BioProject number PRJNA603442, https://www.ncbi.nlm.nih.gov/sra/PRJNA603442.

## Author Contributions

CM and CZ developed the idea and designed the study. CM extracted lipids and analyzed DNA. CM, SC, and JL analyzed lipids. CM, SC, JL, K-UH, and CZ wrote the manuscript.

## Conflict of Interest

The authors declare that the research was conducted in the absence of any commercial or financial relationships that could be construed as a potential conflict of interest.

## Footnotes

1 https://www.i-sanger.com

2 http://rdp.cme.msu.edu/

## References

[B1] Baumann L. M. F. Taubner R.-S. Bauersachs T. Steiner M. Schleper C. Peckmann J. (2018). Intact polar lipid and core lipid inventory of the hydrothermal vent methanogens *Methanocaldococcus villosus* and *Methanothermococcus okinawensis*. *Organ. Geochem.* 126 33–42. 10.1016/j.orggeochem.2018.10.006

[B2] Bayer B. Vojvoda J. Offre P. Alves R. J. E. Elisabeth N. H. Garcia J. A. L. (2015). Physiological and genomic characterization of two novel marine thaumarchaeal strains indicates niche differentiation. *ISME J.* 10 1051–1063. 10.1038/ismej.2015.200 26528837 PMC4839502

[B3] Becker K. W. Lipp J. S. Zhu C. Liu X.-L. Hinrichs K.-U. (2013). An improved method for the analysis of archaeal and bacterial ether core lipids. *Organ. Geochem.* 61 34–44. 10.1016/j.orggeochem.2013.05.007

[B4] Besseling M. A. Hopmans E. C. Bale N. J. Schouten S. Damsté J. S. S. Villanueva L. (2020). The absence of intact polar lipid-derived GDGTs in marine waters dominated by Marine Group II: implications for lipid biosynthesis in Archaea. *Sci. Rep.* 10:294. 10.1038/s41598-019-57035-0 31941956 PMC6962369

[B5] Besseling M. A. Hopmans E. C. Koenen M. van der Meer M. T. J. Vreugdenhil S. Schouten S. (2019). Depth-related differences in archaeal populations impact the isoprenoid tetraether lipid composition of the Mediterranean Sea water column. *Organ. Geochem.* 135 16–31. 10.1016/j.orggeochem.2019.06.008

[B6] Boyd E. S. Pearson A. Pi Y. Li W.-J. Zhang Y. G. He L. (2011). Temperature and pH controls on glycerol dibiphytanyl glycerol tetraether lipid composition in the hyperthermophilic crenarchaeon *Acidilobus sulfurireducens*. *Extremophiles* 15 59–65. 10.1007/s00792-010-0339-y 21125411

[B7] Brochier-Armanet C. Boussau B. Gribaldo S. Forterre P. (2008). Mesophilic crenarchaeota: proposal for a third archaeal phylum, the Thaumarchaeota. *Nat. Rev. Microbiol.* 6 245–252. 10.1038/nrmicro1852 18274537

[B8] Caffrey J. M. Bano N. Kalanetra K. Hollibaugh J. T. (2007). Ammonia oxidation and ammonia-oxidizing bacteria and archaea from estuaries with differing histories of hypoxia. *ISME J.* 1 660–662. 10.1038/ismej.2007.79 18043673

[B9] Cerqueira T. Pinho D. Froufe H. Santos R. S. Bettencourt R. Egas C. (2017). Sediment microbial diversity of three deep-sea hydrothermal vents Southwest of the Azores. *Microb. Ecol.* 74 332–349. 10.1007/s00248-017-0943-9 28144700

[B10] Cole J. R. Wang Q. Cardenas E. Fish J. Chai B. Farris R. J. (2009). The Ribosomal Database Project: improved alignments and new tools for rRNA analysis. *Nucleic Acids Res.* 37 D141–D145. 10.1093/nar/gkn879 19004872 PMC2686447

[B11] de la Torre J. R. Walker C. B. Ingalls A. E. Könneke M. Stahl D. A. (2008). Cultivation of a thermophilic ammonia oxidizing archaeon synthesizing crenarchaeol. *Environ. Microbiol.* 10 810–818. 10.1111/j.1462-2920.2007.01506.x 18205821

[B12] de Rosa M. Gambacorta A. (1988). The lipids of archaebacteria. *Prog. Lipid Res.* 27 153–175. 10.1016/0163-7827(88)90011-2 3151021

[B13] DeLong E. F. (1992). Archaea in coastal marine environments. *Proc. Natl. Acad. Sci. U.S.A.* 89 5685–5689. 10.1073/pnas.89.12.5685 1608980 PMC49357

[B14] Edgar R. C. Haas B. J. Clemente J. C. Quince C. Knight R. (2011). UCHIME improves sensitivity and speed of chimera detection. *Bioinformatics* 27 2194–2200. 10.1093/bioinformatics/btr381 21700674 PMC3150044

[B15] Elling F. J. Könneke M. Lipp J. S. Becker K. W. Gagen E. J. Hinrichs K. U. (2014). Effects of growth phase on the membrane lipid composition of the thaumarchaeon *Nitrosopumilus maritimus* and their implications for archaeal lipid distributions in the marine environment. *Geochim. Cosmochim. Acta* 141 579–597. 10.1016/j.gca.2014.07.005

[B16] Elling F. J. Könneke M. Mußmann M. Greve A. Hinrichs K. U. (2015). Influence of temperature, pH, and salinity on membrane lipid composition and TEX_86_ of marine planktonic thaumarchaeal isolates. *Geochim. Cosmochim. Acta* 171 238–255. 10.1016/j.gca.2015.09.004

[B17] Elling F. J. Könneke M. Nicol G. W. Stieglmeier M. Bayer B. Spieck E. (2017). Chemotaxonomic characterization of the thaumarchaeal lipidome. *Environ. Microbiol.* 19 2681–2700. 10.1111/1462-2920.13759 28419726

[B18] Engelhardt H. (2007). Mechanism of osmoprotection by archaeal S-layers: a theoretical study. *J. Struct. Biol.* 160 190–199. 10.1016/j.jsb.2007.08.004 17888677

[B19] Evans T. W. Könneke M. Lipp J. S. Adhikari R. R. Taubner H. Elvert M. (2018). Lipid biosynthesis of *Nitrosopumilus maritimus* dissected by lipid specific radioisotope probing (lipid-RIP) under contrasting ammonium supply. *Geochim. Cosmochim. Acta* 242 51–63. 10.1016/j.gca.2018.09.001

[B20] Fuhrman J. A. Davis A. A. (1997). Widespread Archaea and novel Bacteria from the deep sea as shown by 16S rRNA gene sequences. *Mar. Ecol. Prog. Ser.* 150 275–285. 10.3354/MEPS150275

[B21] Galand P. E. Gutiérrez-Provecho C. Massana R. Gasol J. M. Casamayor E. O. (2010). Inter-annual recurrence of archaeal assemblages in the coastal NW Mediterranean Sea (Blanes Bay Microbial Observatory). *Limnol. Oceanogr.* 55 2117–2125. 10.4319/lo.2010.55.5.2117

[B22] Haro-Moreno J. M. Rodriguez-Valera F. López-García P. Moreira D. Martin-Cuadrado A. B. (2017). New insights into marine group III Euryarchaeota, from dark to light. *ISME J.* 11 1102–1117. 10.1038/ismej.2016.188 28085158 PMC5437922

[B23] Harvey H. R. Fallon R. D. Patton J. S. (1986). The effect of organic matter and oxygen on the degradation of bacterial membrane lipids in marine sediments. *Geochim. Cosmochim. Acta* 50 795–804. 10.1016/0016-7037(86)90355-8

[B24] He X. Bai Y. Pan D. Chen C.-T. Cheng Q. Wang D. (2013). Satellite views of the seasonal and interannual variability of phytoplankton blooms in the eastern China seas over the past 14 yr (1998–2011). *Biogeosciences* 10 4721–4739. 10.5194/bg-10-4721-2013

[B25] Herfort L. Schouten S. Abbas B. Veldhuis M. J. W. Coolen M. J. L. Wuchter C. (2007). Variations in spatial and temporal distribution of Archaea in the North Sea in relation to environmental variables. *FEMS Microbiol. Ecol.* 62 242–257. 10.1111/j.1574-6941.2007.00397.x 17991018

[B26] Hollibaugh J. T. Gifford S. Sharma S. Bano N. Moran M. A. (2011). Metatranscriptomic analysis of ammonia-oxidizing organisms in an estuarine bacterioplankton assemblage. *ISME J.* 5 866–878. 10.1038/ismej.2010.172 21085199 PMC3105763

[B27] Hopmans E. C. Weijers J. W. H. Schefuß E. Herfort L. Sinninghe Damsté J. S. Schouten S. (2004). A novel proxy for terrestrial organic matter in sediments based on branched and isoprenoid tetraether lipids. *Earth Planet. Sci. Lett.* 224 107–116. 10.1016/j.epsl.2004.05.012

[B28] Huguet C. Hopmans E. C. Febo-Ayala W. Thompson D. H. Damsté J. S. S. Schouten S. (2006). An improved method to determine the absolute abundance of glycerol dibiphytanyl glycerol tetraether lipids. *Organ. Geochem.* 37 1036–1041. 10.1016/j.orggeochem.2006.05.008

[B29] Hurley S. J. Elling F. J. Könneke M. Buchwald C. Wankel S. D. Santoro A. E. (2016). Influence of ammonia oxidation rate on thaumarchaeal lipid composition and the TEX_86_ temperature proxy. *Proc. Natl. Acad. Sci. U.S.A.* 113 7762–7767. 10.1073/pnas.1518534113 27357675 PMC4948339

[B30] Ingalls A. E. (2016). Palaeoceanography: signal from the subsurface. *Nat. Geosci.* 9 572–573. 10.1038/ngeo2765

[B31] Ingalls A. E. Huguet C. Truxal L. T. (2012). Distribution of intact and core membrane lipids of archaeal glycerol dialkyl glycerol tetraethers among size-fractionated particulate organic matter in Hood Canal, Puget Sound. *Appl. Environ. Microbiol.* 78 1480–1490. 10.1128/AEM.07016-11 22226949 PMC3294470

[B32] Iverson V. Morris R. M. Frazar C. D. Berthiaume C. T. Morales R. L. Armbrust E. V. (2012). Untangling genomes from metagenomes: revealing an uncultured class of marine Euryarchaeota. *Science* 335 587–590. 10.1126/science.1212665 22301318

[B33] Karner M. B. DeLong E. F. Karl D. M. (2001). Archaeal dominance in the mesopelagic zone of the Pacific Ocean. *Nature* 409 507–510. 10.1038/35054051 11206545

[B34] Kate M. (1993). “Membrane lipids of archaea,” in *New Comprehensive Biochemistry*, eds Kates M. Kushner D. J. Matheson A. T. (Amsterdam: Elsevier), 261–295. 10.1016/S0167-7306(08)60258-6

[B35] Koga Y. Morii H. (2005). Recent advances in structural research on ether lipids from archaea including comparative and physiological aspects. *Biosci. Biotechnol. Biochem.* 69 2019–2034. 10.1271/bbb.69.2019 16306681

[B36] Koga Y. Nakano M. (2008). A dendrogram of archaea based on lipid component parts composition and its relationship to rRNA phylogeny. *Syst. Appl. Microbiol.* 31 169–182. 10.1016/j.syapm.2008.02.005 18515030

[B37] Könneke M. Bernhard A. E. José R. Walker C. B. Waterbury J. B. Stahl D. A. (2005). Isolation of an autotrophic ammonia-oxidizing marine archaeon. *Nature* 437 543–546. 10.1038/nature03911 16177789

[B38] Könneke M. Schubert D. M. Brown P. C. Hügler M. Standfest S. Schwander T. (2014). Ammonia-oxidizing archaea use the most energy-efficient aerobic pathway for CO_2_ fixation. *Proc. Natl. Acad. Sci. U.S.A.* 111 8239–8244. 10.1073/pnas.1402028111 24843170 PMC4050595

[B39] Lai D. Springstead J. R. Monbouquette H. G. (2008). Effect of growth temperature on ether lipid biochemistry in *Archaeoglobus fulgidus*. *Extremophiles* 12 271–278. 10.1007/s00792-007-0126-6 18157503

[B40] Li M. Baker B. J. Anantharaman K. Jain S. Breier J. A. Dick G. J. (2015). Genomic and transcriptomic evidence for scavenging of diverse organic compounds by widespread deep-sea archaea. *Nat. Commun.* 6:8933. 10.1038/ncomms9933 26573375 PMC4660358

[B41] Lincoln S. A. Wai B. Eppley J. M. Church M. J. Summons R. E. DeLong E. F. (2014). Planktonic euryarchaeota are a significant source of archaeal tetraether lipids in the ocean. *Proc. Natl. Acad. Sci. U.S.A.* 111 9858–9863. 10.1073/pnas.1409439111 24946804 PMC4103328

[B42] Lipp J. S. Hinrichs K.-U. (2009). Structural diversity and fate of intact polar lipids in marine sediments. *Geochim. Cosmochim. Acta* 73 6816–6833. 10.1016/j.gca.2009.08.003

[B43] Liu H. Zhang C. L. Yang C. Chen S. Cao Z. Zhang Z. (2017). Marine group II dominates Planktonic Archaea in Water Column of the Northeastern South China Sea. *Front. Microbiol.* 8:1098. 10.3389/fmicb.2017.01098 28663746 PMC5471323

[B44] Liu X.-L. Lipp J. S. Simpson J. H. Lin Y.-S. Summons R. E. Hinrichs K.-U. (2012). Mono-and dihydroxyl glycerol dibiphytanyl glycerol tetraethers in marine sediments: identification of both core and intact polar lipid forms. *Geochim. Cosmochim. Acta* 89 102–115. 10.1016/j.gca.2012.04.053

[B45] López-García P. Moreira D. López-López A. Rodríguez-Valera F. (2001). A novel haloarchaeal-related lineage is widely distributed in deep oceanic regions. *Environ. Microbiol.* 3 72–78. 10.1046/j.1462-2920.2001.00162.x 11225725

[B46] Martens-Habbena W. Berube P. M. Urakawa H. José R. Stahl D. A. (2009). Ammonia oxidation kinetics determine niche separation of nitrifying Archaea and Bacteria. *Nature* 461 976–979. 10.1038/nature08465 19794413

[B47] Massana R. Murray A. E. Preston C. M. DeLong E. F. (1997). Vertical distribution and phylogenetic characterization of marine planktonic Archaea in the Santa Barbara Channel. *Appl. Environ. Microbiol.* 63 50–56. 8979338 10.1128/aem.63.1.50-56.1997 PMC168301

[B48] Murray A. E. Preston C. M. Massana R. Taylor L. T. Blakis A. Wu K. (1998). Seasonal and spatial variability of bacterial and archaeal assemblages in the coastal waters near Anvers Island, Antarctica. *Appl. Environ. Microbiol.* 64 2585–2595. 9647834 10.1128/aem.64.7.2585-2595.1998 PMC106430

[B49] Nunoura T. Takaki Y. Hirai M. Shimamura S. Makabe A. Koide O. (2015). Hadal biosphere: insight into the microbial ecosystem in the deepest ocean on Earth. *Proc. Natl. Acad. Sci. U.S.A.* 112 E1230–E1236. 10.1073/pnas.1421816112 25713387 PMC4371994

[B50] Pearson A. Pi Y. Zhao W. Li W. Li Y. Inskeep W. (2008). Factors controlling the distribution of archaeal tetraethers in terrestrial hot springs. *Appl. Environ. Microbiol.* 74 3523–3532. 10.1128/AEM.02450-07 18390673 PMC2423032

[B51] Pernthaler A. Preston C. M. Pernthaler J. DeLong E. F. Amann R. (2002). Comparison of fluorescently labeled oligonucleotide and polynucleotide probes for the detection of pelagic marine bacteria and archaea. *Appl. Environ. Microbiol.* 68 661–667. 10.1128/aem.68.2.661-667.2002 11823205 PMC126737

[B52] Pitcher A. Hopmans E. C. Mosier A. C. Park S. J. Rhee S. K. Francis C. A. (2011a). Core and intact polar glycerol dibiphytanyl glycerol tetraether lipids of ammonia-oxidizing Archaea enriched from marine and estuarine sediments. *Appl. Environ. Microbiol.* 77 3468–3477. 10.1128/AEM.02758-10 21441324 PMC3126447

[B53] Pitcher A. Wuchter C. Siedenberg K. Schouten S. Sinninghe Damsté J. S. (2011b). Crenarchaeol tracks winter blooms of ammonia-oxidizing Thaumarchaeota in the coastal North Sea. *Limnol. Oceanogr.* 56 2308–2318. 10.4319/lo.2011.56.6.2308

[B54] Qin W. Amin S. A. Martens-Habbena W. Walker C. B. Urakawa H. Devol A. H. (2014). Marine ammonia-oxidizing archaeal isolates display obligate mixotrophy and wide ecotypic variation. *Proc. Natl. Acad. Sci. U.S.A.* 111 12504–12509. 10.1073/pnas.1324115111 25114236 PMC4151751

[B55] Qin W. Carlson L. T. Armbrust E. V. Devol A. H. Ingalls A. E. (2015). Confounding effects of oxygen and temperature on the TEX_86_ signature of marine Thaumarchaeota. *Proc. Natl. Acad. Sci. U.S.A.* 112, 10979–10984. 10.1073/pnas.1501568112 26283385 PMC4568219

[B56] Quast C. Pruesse E. Yilmaz P. Gerken J. Schweer T. Yarza P. (2013). The SILVA ribosomal RNA gene database project: improved data processing and web-based tools. *Nucleic Acids Res.* 41 D590–D596. 10.1093/nar/gks1219 23193283 PMC3531112

[B57] Rinke C. Rubino F. Messer L. F. Youssef N. Parks D. H. Chuvochina M. (2019). A phylogenomic and ecological analysis of the globally abundant Marine Group II archaea (Ca. Poseidoniales ord. nov.). *ISME J.* 13 663–675. 10.1038/s41396-018-0282-y 30323263 PMC6461757

[B58] Santoro A. E. Dupont C. L. Richter R. A. Craig M. T. Carini P. McIlvin M. R. (2015). Genomic and proteomic characterization of “Candidatus Nitrosopelagicus brevis”: an ammonia-oxidizing archaeon from the open ocean. *Proc. Natl. Acad. Sci. U.S.A.* 112 1173–1178. 10.1073/pnas.1416223112 25587132 PMC4313803

[B59] Santoro A. E. Richter R. A. Dupont C. L. (2019). Planktonic marine archaea. *Annu. Rev. Mar. Sci.* 11 131–158. 10.1146/annurev-marine-121916-063141 30212260

[B60] Schattenhofer M. Fuchs B. M. Amann R. Zubkov M. V. Tarran G. A. Pernthaler J. (2009). Latitudinal distribution of prokaryotic picoplankton populations in the Atlantic Ocean. *Environ. Microbiol.* 11 2078–2093. 10.1111/j.1462-2920.2009.01929.x 19453607

[B61] Schouten S. Hopmans E. C. Baas M. Boumann H. Standfest S. Könneke M. (2008). Intact membrane lipids of “Candidatus *Nitrosopumilus maritimus*,” a cultivated representative of the cosmopolitan mesophilic group I crenarchaeota. *Appl. Environ. Microbiol.* 74 2433–2440. 10.1128/AEM.01709-07 18296531 PMC2293165

[B62] Schouten S. Hopmans E. C. Damsté J. S. S. (2013). The organic geochemistry of glycerol dialkyl glycerol tetraether lipids: a review. *Organ. Geochem.* 54 19–61. 10.1016/j.orggeochem.2012.09.006

[B63] Schouten S. Hopmans E. C. Pancost R. D. Damsté J. S. S. (2000). Widespread occurrence of structurally diverse tetraether membrane lipids: evidence for the ubiquitous presence of low-temperature relatives of hyperthermophiles. *Proc. Natl. Acad. Sci. U.S.A.* 97 14421–14426. 10.1073/pnas.97.26.14421 11121044 PMC18934

[B64] Schouten S. Hopmans E. C. Schefuß E. Damste J. S. S. (2002). Distributional variations in marine crenarchaeotal membrane lipids: a new tool for reconstructing ancient sea water temperatures? *Earth Planet. Sci. Lett.* 204 265–274. 10.1016/S0012-821X(02)00979-2

[B65] Schouten S. Pitcher A. Hopmans E. C. Villanueva L. van Bleijswijk J. Sinninghe Damsté J. S. (2012). Intact polar and core glycerol dibiphytanyl glycerol tetraether lipids in the Arabian Sea oxygen minimum zone: I. Selective preservation and degradation in the water column and consequences for the TEX_86_. *Geochim. Cosmochim. Acta* 98 228–243. 10.1016/j.gca.2012.05.002

[B66] Schouten S. Villanueva L. Hopmans E. C. van Dermeer M. T. J. Damsté J. S. S. (2014). Are Marine Group II Euryarchaeota significant contributors to tetraether lipids in the ocean? *Proc. Natl. Acad. Sci. U.S.A.* 111 E4285. 10.1073/pnas.1416176111 25239232 PMC4205633

[B67] Sinninghe Damsté J. S. Hopmans E. C. Schouten S. van Duin A. C. T. Geenevasen J. A. J. (2002a). Crenarchaeol the characteristic core glycerol dibiphytanyl glycerol tetraether membrane lipid of cosmopolitan pelagic crenarchaeota. *J. Lipid Res.* 43 1641–1651. 10.1194/jlr.m200148-jlr200 12364548

[B68] Sinninghe Damsté J. S. Rijpstra W. I. C. Hopmans E. C. Jung M. Y. Kim J. G. Rhee S. K. (2012). Intact polar and core glycerol dibiphytanyl glycerol tetraether lipids of group I. 1a and I. 1b Thaumarchaeota in soil. *Appl. Environ. Microbiol.* 78 6866–6874. 10.1128/AEM.01681-12 22820324 PMC3457472

[B69] Sinninghe Damst J. S. Rijpstra W. I. Hopmans E. C. Prahl F. G. Wakeham S. G. Schouten S. (2002b). Distribution of membrane lipids of planktonic Crenarchaeota in the Arabian Sea. *Appl. Environ. Microbiol.* 68 2997–3002. 10.1128/aem.68.6.2997-3002.2002 12039760 PMC123986

[B70] Sintes E. de Corte D. Ouillon N. Herndl G. J. (2015). Macroecological patterns of archaeal ammonia oxidizers in the Atlantic Ocean. *Mol. Ecol.* 24 4931–4942. 10.1111/mec.13365 26336038 PMC4950044

[B71] Sollai M. Villanueva L. Hopmans E. C. Keil R. G. Sinninghe Damste J. S. (2019). Archaeal sources of intact membrane lipid biomarkers in the oxygen deficient zone of the eastern tropical South Pacific. *Front. Microbiol.* 10:765. 10.3389/fmicb.2019.00765 31031734 PMC6470261

[B72] Spang A. Hatzenpichler R. Brochier-Armanet C. Rattei T. Tischler P. Spieck E. (2010). Distinct gene set in two different lineages of ammonia-oxidizing archaea supports the phylum Thaumarchaeota. *Trends Microbiol.* 18 331–340. 10.1016/J.TIM.2010.06.003 20598889

[B73] Stahl D. A. (1991). “Development and application of nucleic acid probes,” in *Nucleic Acid Techniques in Bacterial Systematics*, eds Stackebrandt E. Goodfellow M. (Chichester: John Wiley & Sons Ltd), 205–248.

[B74] Stahl D. A. de la Torre J. R. (2012). Physiology and diversity of ammonia-oxidizing archaea. *Annu. Rev. Microbiol.* 66 83–101. 10.1146/annurev-micro-092611-150128 22994489

[B75] Sturt H. F. Summons R. E. Smith K. Elvert M. Hinrichs K. (2004). Intact polar membrane lipids in prokaryotes and sediments deciphered by high-performance liquid chromatography/electrospray ionization multistage mass spectrometry—new biomarkers for biogeochemistry and microbial ecology. *Rapid Commun. Mass Spectr.* 18 617–628. 10.1002/rcm.1378 15052572

[B76] Teira E. Lebaron P. van Aken H. Herndl G. J. (2006). Distribution and activity of Bacteria and Archaea in the deep water masses of the North Atlantic. *Limnol. Oceanogr.* 51 2131–2144. 10.4319/lo.2006.51.5.2131

[B77] Tian J. Fan L. Liu H. Liu J. Li Y. Qin Q. (2018). A nearly uniform distributional pattern of heterotrophic bacteria in the Mariana Trench interior. *Deep Sea Res. Part I Oceanogr. Res. Pap.* 142 116–126. 10.1016/j.dsr.2018.10.002

[B78] Tierney J. E. Tingley M. P. (2015). A TEX_86_ surface sediment database and extended Bayesian calibration. *Sci. Data* 2:150029. 10.1038/sdata.2015.29 26110065 PMC4477698

[B79] Tully B. J. (2019). Metabolic diversity within the globally abundant Marine Group II Euryarchaea offers insight into ecological patterns. *Nat. Commun.* 10:271. 10.1038/s41467-018-07840-4 30655514 PMC6336850

[B80] Turich C. Freeman K. H. Bruns M. A. Conte M. Jones A. D. Wakeham S. G. (2007). Lipids of marine Archaea: patterns and provenance in the water-column and sediments. *Geochim. Cosmochim. Acta* 71 3272–3291. 10.1016/j.gca.2007.04.013

[B81] Uda I. Sugai A. Itoh Y. H. Itoh T. (2001). Variation in molecular species of polar lipids from *Thermoplasma acidophilum* depends on growth temperature. *Lipids* 36 103–105. 10.1007/s11745-001-0914-2 11214723

[B82] Uda I. Sugai A. Itoh Y. H. Itoh T. (2004). Variation in molecular species of core lipids from the order *Thermoplasmales* strains depends on the growth temperature. *J. Oleo Sci.* 53 399–404. 10.5650/jos.53.399

[B83] Villanueva L. Schouten S. Sinninghe Damsté J. S. (2016). Phylogenomic analysis of lipid biosynthetic genes of Archaea shed light on the “lipid divide”. *Environ. Microbiol.* 19 54–69. 10.1111/1462-2920.13361 27112361

[B84] Wang J. X. Wei Y. Wang P. Hong Y. Zhang C. L. (2015). Unusually low TEX_86_ values in the transitional zone between Pearl River estuary and coastal South China Sea: impact of changing archaeal community composition. *Chem. Geol.* 402 18–29. 10.1016/j.chemgeo.2015.03.002

[B85] White D. C. Davis W. M. Nickels J. S. King J. D. Bobbie R. J. (1979). Determination of the sedimentary microbial biomass by extractible lipid phosphate. *Oecologia* 40 51–62. 10.1007/BF00388810 28309603

[B86] Wörmer L. Lipp J. S. Schröder J. M. Hinrichs K.-U. (2013). Application of two new LC–ESI–MS methods for improved detection of intact polar lipids (IPLs) in environmental samples. *Organ. Geochem.* 59 10–21. 10.1016/j.orggeochem.2013.03.004

[B87] Wuchter C. (2006). *Ecology and Membrane Lipid Distribution of Marine Crenarchaeota: Implications for TEX_86_ paleothermometry*, dissertation Utrecht University, Utrecht Available online at: https://dspace.library. uu.nl/bitstream/handle/1874/8653/index.htm;jsessionid=2B9F9E159D41A76C 2561D5B56F7E6511?sequence=5

[B88] Xie W. Luo H. Murugapiran S. K. Dodsworth J. A. Chen S. Sun Y. (2018). Localized high abundance of Marine Group II archaea in the subtropical Pearl River Estuary: implications for their niche adaptation. *Environ. Microbiol.* 20 734–754. 10.1111/1462-2920.14004 29235710

[B89] Xie W. Zhang C. Zhou X. Wang P. (2014). Salinity-dominated change in community structure and ecological function of Archaea from the lower Pearl River to coastal South China Sea. *Appl. Microbiol. Biotechnol.* 98 7971–7982. 10.1007/s00253-014-5838-9 24880629

[B90] Yu Y. Lee C. Kim J. Hwang S. (2005). Group-specific primer and probe sets to detect methanogenic communities using quantitative real-time polymerase chain reaction. *Biotechnol. Bioeng.* 89 670–679. 10.1002/bit.20347 15696537

[B91] Zeng Z. Liu X.-L. Farley K. R. Wei J. H. Metcalf W. W. Summons R. E. (2019). GDGT cyclization proteins identify the dominant archaeal sources of tetraether lipids in the ocean. *Proc. Natl. Acad. Sci. U.S.A.* 116 22505–22511. 10.1073/pnas.1909306116 31591189 PMC6842593

[B92] Zhang C. L. Wang J. Dodsworth J. A. Williams A. J. Zhu C. Hinrichs K.-U. (2013). In situ production of branched glycerol dialkyl glycerol tetraethers in a great basin hot spring (USA). *Front. Microbiol.* 4:181. 10.3389/fmicb.2013.00181 23847605 PMC3705189

[B93] Zhang C. L. Xie W. Martin-Cuadrado A.-B. Rodriguez-Valera F. (2015). Marine Group II Archaea, potentially important players in the global ocean carbon cycle. *Front. Microbiol.* 6:1108. 10.3389/fmicb.2015.01108 26528260 PMC4602124

[B94] Zhang Y. G. Pagani M. Wang Z. (2016). Ring Index: a new strategy to evaluate the integrity of TEX_86_ paleothermometry. *Paleoceanography* 31 220–232. 10.1002/2015PA002848

[B95] Zhang Y. G. Zhang C. L. Liu X. L. Li L. Hinrichs K. U. Noakes J. E. (2011). Methane Index: a tetraether archaeal lipid biomarker indicator for detecting the instability of marine gas hydrates. *Earth Planet. Sci. Lett.* 307 525–534. 10.1016/j.epsl.2011.05.031

[B96] Zhu C. Lipp J. S. Wörmer L. Becker K. W. Schröder J. Hinrichs K. U. (2013). Comprehensive glycerol ether lipid fingerprints through a novel reversed phase liquid chromatography-mass spectrometry protocol. *Organ. Geochem.* 65 53–62. 10.1016/j.orggeochem.2013.09.012

[B97] Zhu C. Wakeham S. G. Elling F. J. Basse A. Mollenhauer G. Versteegh G. J. M. (2016). Stratification of archaeal membrane lipids in the ocean and implications for adaptation and chemotaxonomy of planktonic archaea. *Environ. Microbiol.* 18 4324–4336. 10.1111/1462-2920.13289 26950522

[B98] Zhu C. Yoshinaga M. Y. Peters C. A. Liu X. L. Elvert M. Hinrichs K. U. (2014). Identification and significance of unsaturated archaeal tetraether lipids in marine sediments. *Rapid Commun. Mass Spectr.* 28 1144–1152. 10.1002/rcm.6887 24711277

