# Supplementary material for: Marine Group II Euryarchaeota Contribute to the Archaeal Lipid Pool in Northwestern Pacific Ocean Surface Waters
Source: Front Microbiol. 2020 Jun 5;11:1034. doi: 10.3389/fmicb.2020.01034 (PMC7291766; doi:10.3389/fmicb.2020.01034)
Supplement: Supplementary file 1 [file Data_Sheet_1.docx]

Supplementary Material

**Fig. S1.** Chemical structures of glycerol dibiphytanyl glycerol tetraethers (GDGTs) and glycerol diphytanyl diethers (archaeols; ARs) detected in this study. Intact polar lipids mentioned in the text consist of one or two glycosidic or glycophosphatidic head groups attached to the glycerol of a diether or tetraether core lipid. GDGTs may contain up to three cyclopentane rings or one cyclohexane and four cyclopentane rings (crenarchaeol). GDGTs may also contain additional hydroxyl groups. ARs may contain a methoxy group at the *sn*-1 position of the glycerol moiety (MeO-AR). We also detected unsaturated archaeols (C-uns-AR (u), where u = the number of double bond equivalents, which means double bonds or rings). In the figure, C-uns-AR (4) is represented where, in P_m:n_, m = the number of cycloalkyl moieties, and n = the number of double bonds (Zhu et al., 2013).

**Fig. S2.** Archaeal community structure expressed by the relative abundance of archaeal 16S rRNA gene sequences. Stations E-3 and E-6 do not show data because of failed amplification. Blue labels are the N-samples and orange labels are the E-samples.

**Fig. S3.** Phylogenetic tree based on Archaeal 16S rRNA gene sequencing (labels marked in blue represent major OTUs from the N-samples; labels in orange represent major OTUs from the E-samples). Percentage in bold represents the percentage of OTU 16S rRNA gene amplicon reads over the total reads.

**Fig. S4.** Plots of MG I cell density against: a) C-uns-ARs; b) MeO-AR. Blue squares = N-samples; orange dots = E-samples. The only significant correlation in 4a is represented with a dashed line (R^2^ > 0.5 and *p* < 0.05) and does not include sample E-7 (in the upper left corner). The line in 4b shows a weak and not significant (*p* > 0.05) correlation.

**Table S1.** Description of the sampling conditions and oceanographic settings.

**Table S2.** Parameters of sMRM method for specific archaeal lipids (CE: collision energy, arbitrary units).

**Table S3.** Relative abundance of GDGT core structure for each polar headgroup detected.

**Table S4.** Significant Pearson correlation coefficients (*p* value < 0.05) between archaeal cell densities of total archaea based on qPCR quantification, MG I and MG II inferred by archaeal community composition from sequencing, and tetraether lipid abundances.

**Table S5.** Relative abundance of C-uns-ARs and the Unsaturation Index (UI) of C-uns-ARs. UI was calculated as the weighted mean where the number of double bond equivalents of each compound is weighted by its relative proportion.

**Table S6.** Ring index values for each GDGT pool bearing a different polar headgroup.

**Fig. S1**

**
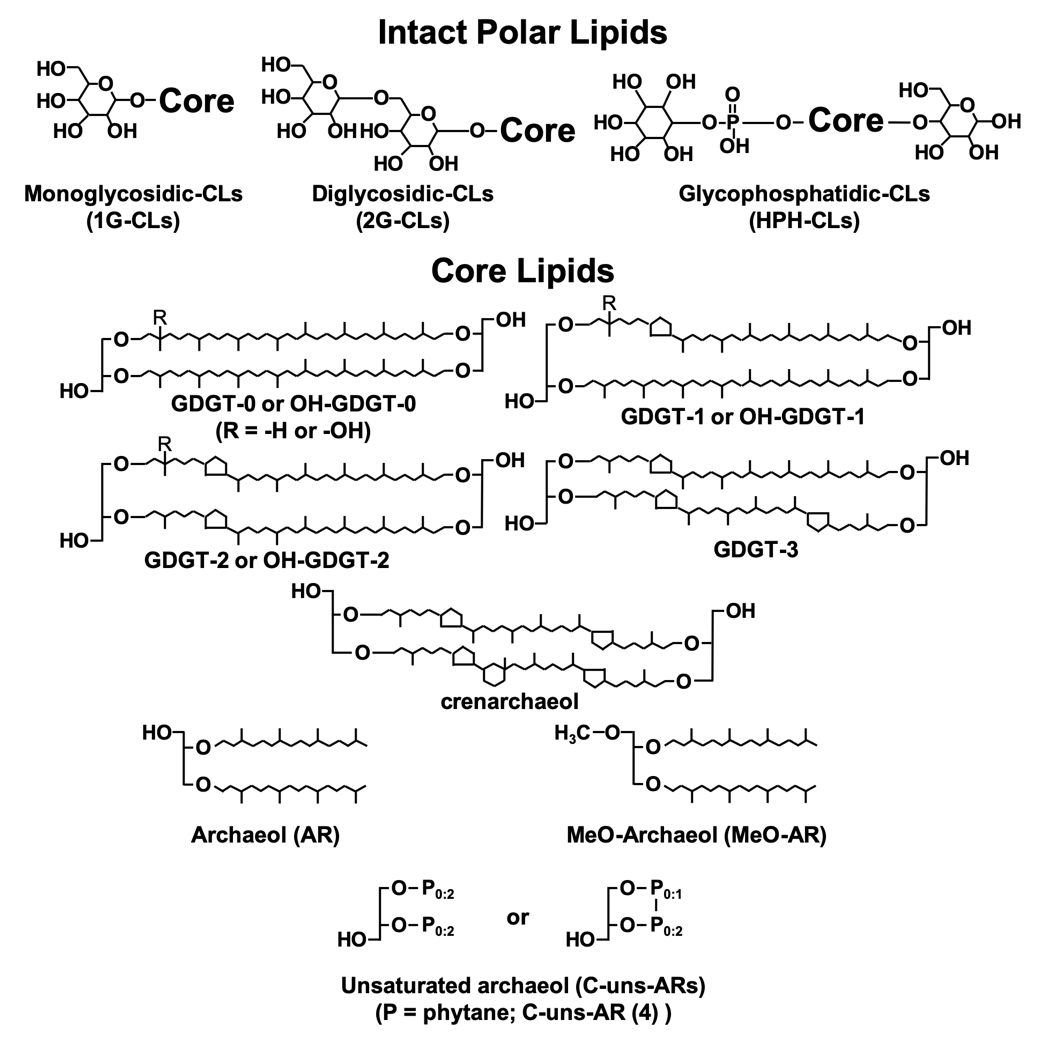
**

**Fig. S2**


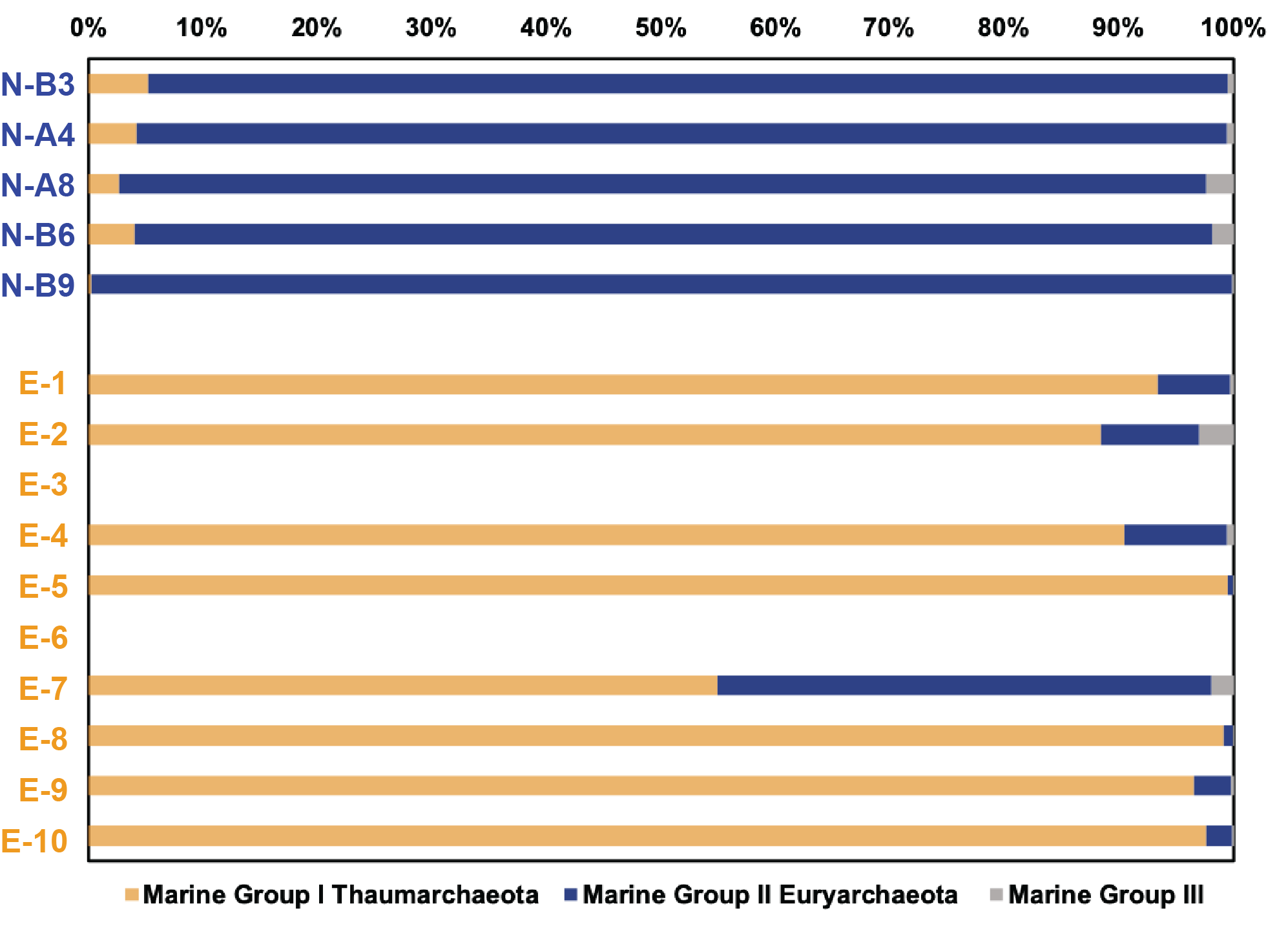


**Fig. S3**


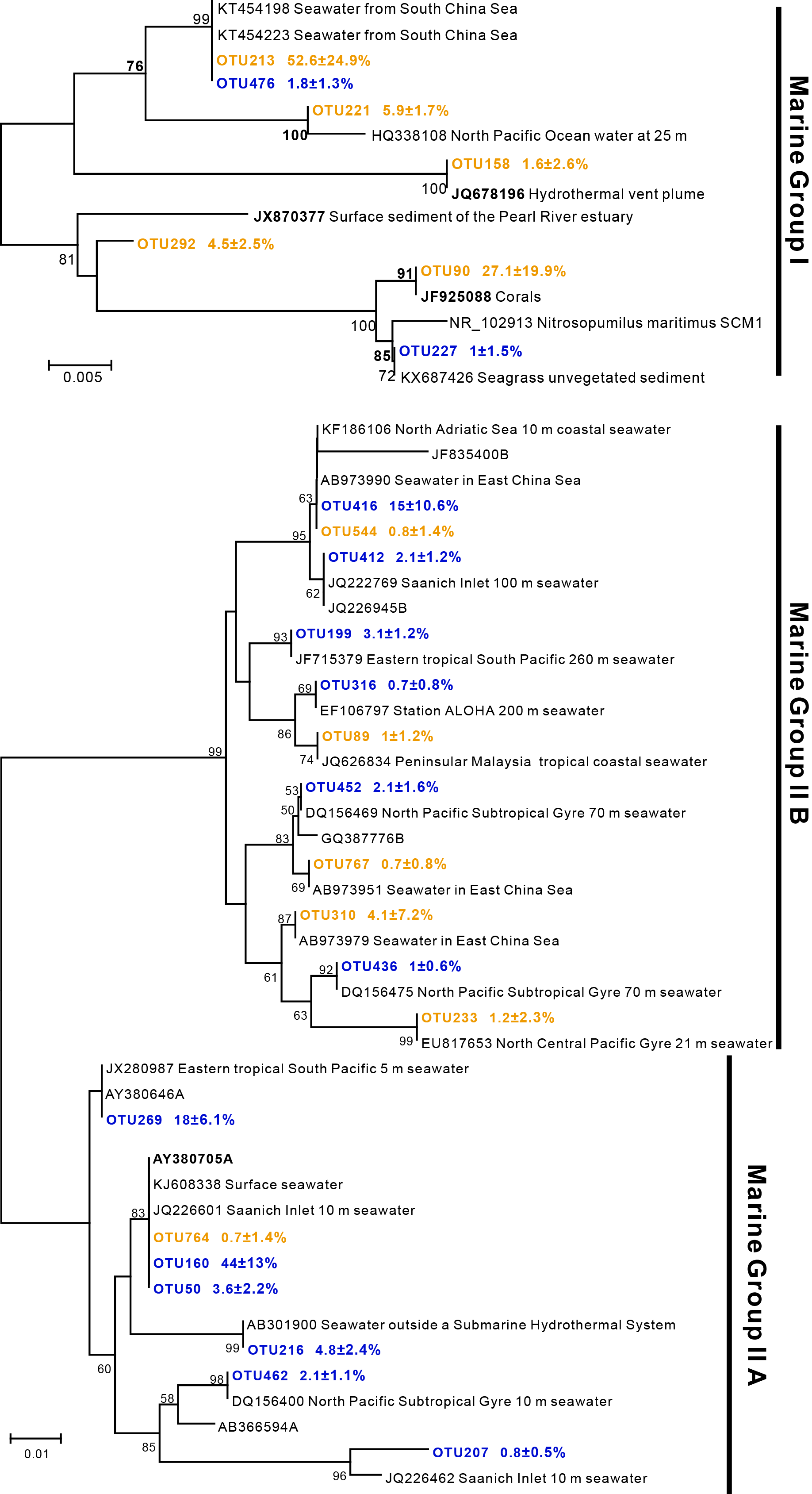


**Fig. S4**


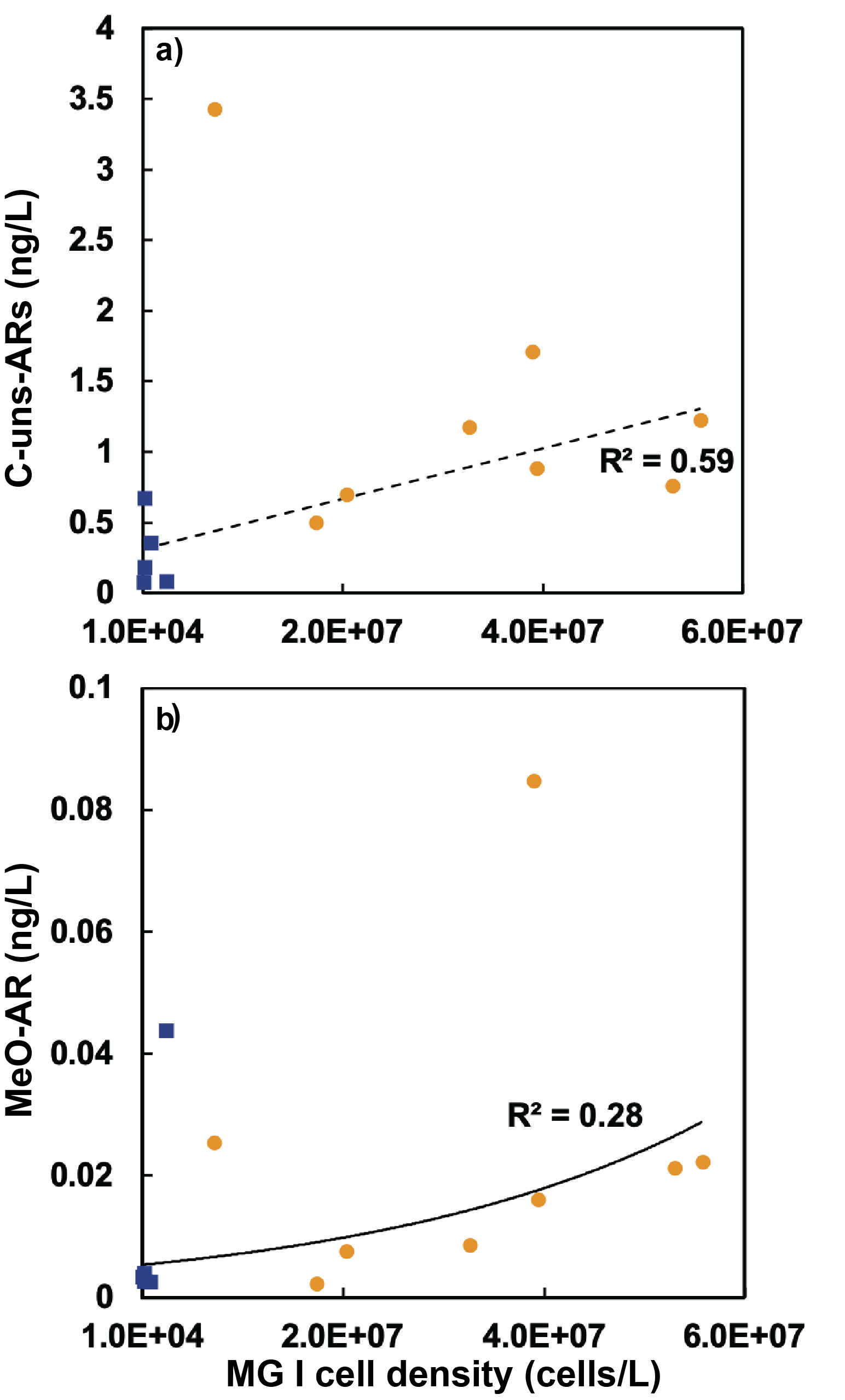


**Table S1**

| Site | Location (N) | Location (E) | Water depth | Sampling depth | Filtered seawater | T | Salinity | NO_3_^-^ | NO_2_^-^ | PO_4_^3-^ | SiO_3_^2-^ |
| --- | --- | --- | --- | --- | --- | --- | --- | --- | --- | --- | --- |
|  | (˚) | (˚) | (m) | (mbsl) | (L) | (˚C) | (PSU) | (µmol/L) | (µmol/L) | (µmol/L) | (µmol/L) |
| N-B3 | 36 | 147 | 5600 | 2 to 5 | 100 | 18.2 | 34.7 | 4.97 | 0.27 | 0.20 | 4.68 |
| N-A4 | 34 | 147.83 | 5800 | 2 to 5 | 80 | 17.5 | 34.6 | 8.84 | 0.25 | 0.16 | 4.12 |
| N-A8 | 34.01 | 152 | 5500 | 2 to 5 | 125 | 18.0 | 34.8 | 4.63 | 0.23 | 0.10 | 1.80 |
| N-B6 | 32.6 | 146.6 | 5886 | 2 to 5 | 100 | 17.9 | 34.8 | 1.73 | 0.05 | 0.05 | 1.53 |
| N-B9 | 30 | 147 | 6265 | 2 to 5 | 200 | 18.7 | 34.9 | 0.77 | 0.02 | 0.03 | 0.11 |
|  |  |  |  |  |  |  |  |  |  |  |  |
| E-1 | 29.46 | 127.96 | 982 | 9.6 | 95 | 26.4 | 34.6 | 0.90 | 0.01 | 0.03 | 0.55 |
| E-2 | 30.65 | 127.06 | 131 | 9.6 | 95 | 25.0 | 34.4 | 1.11 | 0.02 | 0.03 | 1.14 |
| E-3 | 30.59 | 128.19 | 481 | 10.2 | 95 | 24.3 | 34.4 | 1.08 | 0.02 | 0.08 | 1.57 |
| E-4 | 30.45 | 128.58 | 802 | 9.4 | 95 | 24.6 | 34.4 | 0.53 | 0.01 | 0.03 | 0.24 |
| E-5 | 28.92 | 127.05 | 247 | 9.6 | 95 | 25.0 | 34.2 | 0.85 | 0.05 | 0.04 | 0.51 |
| E-6 | 28.89 | 127.15 | 462 | 10.2 | 95 | 25.1 | 34.2 | 0.61 | 0.04 | 0.07 | 0.75 |
| E-7 | 28.86 | 127.25 | 748 | 10.2 | 95 | 25.0 | 34.2 | 1.05 | 0.03 | 0.06 | 1.48 |
| E-8 | 28.81 | 127.39 | 961 | 10.1 | 95 | 26.0 | 34.7 | 2.05 | 0.14 | 0.06 | 1.46 |
| E-9 | 28.76 | 127.54 | 997 | 9.6 | 95 | 26.3 | 34.7 | 0.87 | 0.01 | 0.04 | 0.78 |
| E-10 | 28.70 | 127.74 | 1136 | 7.6 | 95 | 26.1 | 34.7 | 1.91 | 0.02 | 0.05 | 1.06 |

**Table S2**

| Compounds | CE | Precursor ion [M+NH_4_]^+^ | Product ion m/z |
| --- | --- | --- | --- |
| HPH-GDGTs | 45 | 1723.3-1713.3 | 1544.3-1534.3 |
| 2G-OH-GDGTs | 50 | 1659.4-1651.4 | 1300.3-1292.3 |
| 2G-GDGTs | 47 | 1643.4-1633.4 | 1302.2-1292.3 |
| 1G-OH-GDGTs | 40 | 1497.4-1493.4 | 1300.3-1292.3 |
| 1G-GDGTs | 40 | 1481.4-1471.4 | 1302.2-1292.3 |
| C-GDGTs | 15 | 1319.3-1309.3 | 1319.3-1309.3 |
| 2G-AR | 30 | 994.8 | 653.7 |
| 1G-AR | 25 | 832.8 | 653.7 |
| C-AR | 25 | 670.7 | 373.7 |
| C-uns-AR (1) | 15 | 668.7 | 279.3 |
| C-uns-AR (2) | 15 | 666.7 | 279.3; 277.3 |
| C-uns-AR (3) | 20 | 664.7 | 277.3; 275.3 |
| C-uns-AR (4) | 20 | 662.7 | 277.3; 275.3; 273.3 |
| C-uns-AR (5) | 20 | 660.7 | 275.3; 273.3 |
| C-uns-AR (6) | 20 | 658.7 | 275.3; 273.3 |
| C-uns-AR (7) | 25 | 656.7 | 273.3 |
| MeO-AR | 25 | 667.7 | 387.4 |

**Table S3**

|  | Relative abundance (%) | | | | | | | | | | | | | | | | | | | | | | |
| --- | --- | --- | --- | --- | --- | --- | --- | --- | --- | --- | --- | --- | --- | --- | --- | --- | --- | --- | --- | --- | --- | --- | --- |
|  | HPH-GDGTs | | | | |  | 2G-GDGTs | | | | |  | 1G-GDGTs | | | | |  | C-GDGTs | | | | |
| Site | GDGT-0 | GDGT-1 | GDGT-2 | GDGT-3 | crenarchaeol |  | GDGT-0 | GDGT-1 | GDGT-2 | GDGT-3 | crenarchaeol |  | GDGT-0 | GDGT-1 | GDGT-2 | GDGT-3 | crenarchaeol |  | GDGT-0 | GDGT-1 | GDGT-2 | GDGT-3 | crenarchaeol |
| N-B3 | 53.4 | 6.8 | 0 | 0 | 39.8 |  | 2.5 | 18.5 | 36.8 | 30.4 | 11.8 |  | 53.5 | 7.4 | 3.4 | 0.7 | 34.9 |  | 40.7 | 6.5 | 3.3 | 1.0 | 48.4 |
| N-A4 | 43 | 0 | 0 | 0 | 57.0 |  | 1.9 | 9.9 | 36.7 | 31.6 | 19.9 |  | 46.3 | 10.8 | 4.1 | 0.8 | 38.0 |  | 39.2 | 9.7 | 5.8 | 0.8 | 44.5 |
| N-A8 | 40.1 | 9.4 | 2.5 | 0 | 47.9 |  | 1.7 | 11.0 | 36.8 | 29.1 | 21.3 |  | 52.1 | 12.0 | 7.0 | 1.5 | 27.5 |  | 36.6 | 8.5 | 6.9 | 1.7 | 46.2 |
| N-B6 | 37.9 | 8.6 | 2.7 | 0 | 50.8 |  | 1.4 | 8.17 | 34.6 | 30.9 | 24.9 |  | 50.5 | 11.9 | 5.6 | 0.9 | 31.2 |  | 32.9 | 7.4 | 6.7 | 2.0 | 51.1 |
| N-B9 | 100 | 0 | 0 | 0 | 0 |  | 0 | 8.0 | 36.4 | 33.9 | 21.7 |  | 39.0 | 11.4 | 7.1 | 2.2 | 40.4 |  | 35.1 | 8.4 | 6.3 | 0.0 | 50.1 |
|  |  |  |  |  |  |  |  |  |  |  |  |  |  |  |  |  |  |  |  |  |  |  |  |
| E-1 | 17.3 | 11.9 | 10.5 | 0 | 60.3 |  | 0 | 4.8 | 37.8 | 26.3 | 31.1 |  | 18.0 | 10.5 | 12.1 | 1.8 | 57.6 |  | 17.2 | 7.8 | 10.5 | 2.4 | 62.0 |
| E-2 | 17.5 | 14.4 | 9.6 | 0 | 58.4 |  | 0 | 7.0 | 44.2 | 25.8 | 23.1 |  | 19.0 | 10.3 | 10.5 | 1.4 | 58.8 |  | 13.9 | 8.2 | 10.8 | 2.8 | 64.2 |
| E-3 | 26.3 | 14.8 | 5.9 | 0 | 53.0 |  | 0.7 | 5.8 | 33.2 | 33.8 | 26.6 |  | 27.8 | 13.1 | 6.7 | 1.0 | 51.4 |  | 21.9 | 9.0 | 7.3 | 2.8 | 59.0 |
| E-4 | 19.7 | 15 | 8.1 | 0 | 57.3 |  | 0 | 6.5 | 41.4 | 29.0 | 23.0 |  | 18.6 | 11.3 | 10.5 | 1.9 | 57.8 |  | 15.0 | 7.4 | 10.0 | 3.9 | 63.6 |
| E-5 | 100 | 0 | 0 | 0 | 0 |  | 0 | 4.2 | 38.9 | 31.5 | 25.5 |  | 14.5 | 10.0 | 11.6 | 1.6 | 62.4 |  | 14.0 | 8.3 | 12.3 | 3.5 | 61.9 |
| E-6 | 31.2 | 14 | 5.2 | 0 | 49.5 |  | 0 | 6.4 | 37.4 | 35.2 | 21.0 |  | 22.2 | 10.2 | 6.5 | 1.1 | 59.9 |  | 19.1 | 8.4 | 8.1 | 3.3 | 61.1 |
| E-7 | 28.3 | 12.6 | 4.2 | 0 | 54.9 |  | 0.5 | 7.4 | 39.8 | 32.1 | 20.3 |  | 31.0 | 12.0 | 5.4 | 0.8 | 50.9 |  | 20.9 | 9.2 | 7.5 | 2.3 | 60.1 |
| E-8 | 13.1 | 8 | 0 | 0 | 78.8 |  | 0.9 | 12.0 | 44.8 | 26.7 | 15.7 |  | 26.3 | 10.1 | 4.0 | 0.7 | 58.9 |  | 21.1 | 8.6 | 5.7 | 2.6 | 62.0 |
| E-9 | 17.3 | 0 | 0 | 0 | 82.7 |  | 0.6 | 5.3 | 40.1 | 28.8 | 25.2 |  | 16.1 | 9.1 | 7.5 | 1.0 | 66.2 |  | 14.3 | 7.6 | 9.2 | 3.2 | 65.8 |
| E-10 | 12.9 | 13.8 | 9.3 | 0 | 64.0 |  | 0.5 | 2.0 | 36.1 | 27.5 | 33.9 |  | 12.4 | 7.8 | 12.6 | 1.6 | 65.7 |  | 12.4 | 7.7 | 13.9 | 3.5 | 62.5 |

**Table S4**

| Archaea | Lipid | R value |
| --- | --- | --- |
| Total Archaea | 1G-GDGT-2 | 0.59 |
| Total Archaea | C-GDGT-2 | 0.71 |
| Total Archaea | C-GDGT-3 | 0.64 |
| Total Archaea | C-crenarchaeol | 0.52 |
| MG I | HPH-GDGT-0 | -0.67 |
| MG I | 1G-OH-GDGT-2 | -0.68 |
| MG I | 1G-GDGT-2 | 0.69 |
| MG I | 1G-GDGT-3 | 0.59 |
| MG I | C-GDGT-2 | 0.59 |
| MG I | C-GDGT-3 | 0.63 |
| MG II | HPH-GDGT-0 | 0.57 |
| MG II | 2G-OH-GDGT-0 | 0.89 |
| MG II | 2G-OH-GDGT-1 | 0.59 |
| MG II | 2G-GDGT-0 | 0.67 |
| MG II | 2G-GDGT-1 | 0.59 |
| MG II | 1G-OH-GDGT-1 | 0.85 |
| MG II | C-GDGT-0 | 0.89 |
| MG II | C-GDGT-1 | 0.69 |
| MG II | C-crenarchaeol | 0.67 |

**Table S5**

|  | Relative abundance (%) | | | | | | |  | UI-uns-ARs |
| --- | --- | --- | --- | --- | --- | --- | --- | --- | --- |
| Site | C-uns-AR (1) | C-uns-AR (2) | C-uns-AR (3) | C-uns-AR (4) | C-uns-AR (5) | C-uns-AR (6) | C-uns-AR (7) |  |  |
| N-B3 | 0 | 0 | 1.6 | 83.6 | 7.8 | 7.0 | 0.0 |  | 4.2 |
| N-A4 | 0 | 0.1 | 0.7 | 39.6 | 16.2 | 42.5 | 0.9 |  | 5.0 |
| N-A8 | 0 | 0 | 4.4 | 68.2 | 11.5 | 15.2 | 0.6 |  | 4.4 |
| N-B6 | 0 | 0.1 | 1.5 | 59.0 | 12.6 | 26.0 | 0.8 |  | 4.7 |
| N-B9 | 0 | 0 | 0 | 91.0 | 7.4 | 1.6 | 0.0 |  | 4.1 |
|  |  |  |  |  |  |  |  |  |  |
| E-1 | 0.007 | 1.0 | 6.0 | 81.3 | 6.2 | 5.2 | 0.2 |  | 4.1 |
| E-2 | 0.004 | 0.9 | 5.1 | 72.3 | 8.4 | 13.0 | 0.3 |  | 4.3 |
| E-3 | 0 | 0.2 | 2.3 | 54.7 | 13.8 | 28.0 | 1.0 |  | 4.7 |
| E-4 | 0.004 | 1.0 | 6.1 | 75.7 | 7.1 | 9.8 | 0.3 |  | 4.2 |
| E-5 | 0.011 | 1.4 | 6.8 | 76.8 | 7.0 | 7.6 | 0.4 |  | 4.1 |
| E-6 | 0 | 0.5 | 4.5 | 68.0 | 12.3 | 14.2 | 0.5 |  | 4.4 |
| E-7 | 0 | 0.4 | 4.1 | 63.4 | 12.8 | 18.8 | 0.6 |  | 4.5 |
| E-8 | 0.004 | 0.7 | 4.3 | 72.9 | 9.1 | 12.6 | 0.4 |  | 4.3 |
| E-9 | 0.012 | 1.1 | 6.0 | 73.3 | 10.2 | 9.1 | 0.3 |  | 4.2 |
| E-10 | 0.035 | 1.7 | 7.5 | 79.1 | 6.4 | 5.2 | 0.2 |  | 4.1 |

**Table S6**

| Site | Ring Index | | | |
| --- | --- | --- | --- | --- |
|  | HPH | 2G | 1G | C |
| N-B3 | 1.7 | 2.3 | 1.6 | 2.1 |
| N-A4 | 2.3 | 2.6 | 1.7 | 2.0 |
| N-A8 | 2.1 | 2.6 | 1.4 | 2.1 |
| N-B6 | 2.2 | 2.7 | 1.5 | 2.3 |
| N-B9 | 0.0 | 2.7 | 1.9 | 2.2 |
|  |  |  |  |  |
| E-1 | 2.7 | 2.8 | 2.7 | 2.8 |
| E-2 | 2.7 | 2.6 | 2.7 | 3.0 |
| E-3 | 2.4 | 2.8 | 2.3 | 2.7 |
| E-4 | 2.6 | 2.7 | 2.7 | 2.9 |
| E-5 | 0.0 | 2.8 | 2.9 | 2.9 |
| E-6 | 2.2 | 2.7 | 2.7 | 2.8 |
| E-7 | 2.4 | 2.6 | 2.3 | 2.7 |
| E-8 | 3.2 | 2.4 | 2.6 | 2.8 |
| E-9 | 3.3 | 2.7 | 2.9 | 3.0 |
| E-10 | 2.9 | 2.9 | 3.0 | 3.0 |
